# Quantifying the utility of type 2 diabetes polygenic risk score for predicting incident diabetes: an analysis of large US-based cohort studies

**DOI:** 10.1186/s12920-026-02386-7

**Published:** 2026-05-12

**Authors:** Robert L Hanson, Sayuko Kobes, Lauren E Wedekind, Elsa Vazquez Arreola, Maria Jose Ramirez-Luzuriaga, Wen-Chi Hsueh

**Affiliations:** 1 Phoenix Epidemiology and Clinical Research Branch, National Institute of Diabetes and Digestive and Kidney Diseases Phoenix, Arizona, 85004 USA; 2 Phoenix Epidemiology and Clinical Research Branch, National Institute of Diabetes and Digestive and Kidney Diseases, 445 N 5th Street, Suite 210, Phoenix, AZ 85004 USA

**Keywords:** Type 2 diabetes, Prediction, Polygenic risk score

## Abstract

**Background:**

Genome-wide association analyses (GWASs) have identified numerous genetic variants associated with type 2 diabetes, but the utility of polygenic risk scores (PRSs) derived from these associations for predicting future incident diabetes remains uncertain. We analyzed utility of PRSs to predict incident diabetes in longitudinal studies, consisting of African Americans (AfrAm) and European Americans (EurAm) from the publicly available dbGAP resource.

**Methods:**

Data consisted of 3,886 AfrAm and 17,345 EurAm with GWAS data, who were initially without diabetes; there were 688 incident diabetes cases in AfrAm and 2,304 in EurAm. Strength of association was assesed by the hazard ratio (HR), while ability to discriminate between those who did and did not develop diabetes was assessed by change in the C statistic (ΔC), and ability to correctly reclassify diabetes risk was assessed by the net reclassficiation information (NRI). Decision curve analysis was used to model potential benefits of using the PRS to select individuals for a preventive intevention across a range of thresholds for implementation.

**Results:**

Among the 8 PRSs evaluated, PGS002308 (1.2 M variants, from a multi-ancestry GWAS) provided the best performance. With adjustment for age, sex, parental history of diabetes, body mass index (BMI) and fasting glucose levels, the HR was 1.50 per 5000 risk alleles (95% confidence interval, 1.41–1.60). The NRI was 0.313 (0.229–0.397) and ΔC was 0.010 (0.007–0.014). In comparison, NRI was 0.401 (0.338–0.464) for BMI and 0.257 (0.188–0.327) for serum lipid levels. The PRS provided a 5.9% (1.6–10.4) improvement in area under the decision curve. For most measures of predictive utility there was little heterogeneity between AfrAm and EurAm. In contrast, measures of predictive utility were stronger in younger, than in older, individuals.

**Conclusions:**

PRSs for type 2 diabetes, as currently constructed, provide utility for clinical prevention purposes that is similar to that provided by commonly used clinical predictors; utility is likely greater in younger than in older individuals. Further studies of diabetes risk assessment and those that aim to determine optimal target populations for diabetes prevention efforts would be strengthened by incorporating PRSs.

**Supplementary Information:**

The online version contains supplementary material available at 10.1186/s12920-026-02386-7.

## Introduction

Genome-wide association studies (GWASs) have identified a large number of variants associated with type 2 diabetes [1–5]. There is considerable interest in the use of polygenic risk scores (PRSs) derived from these GWASs for clinical purposes. One such potential use is for clinical prevention: i.e., to select individuals at high risk for developing diabetes for preventive interventions. While most studies of associations between PRSs and type 2 diabetes have been cross-sectional [4–9], assessment of the utility for clinical prevention optimally requires analysis of diabetes incidence in individuals who are initially without diabetes. There have been some studies of the extent to which type 2 diabetes PRSs predict incident diabetes [10–20]. These have generally shown that PRSs strongly associate with incident diabetes, but the extent to which they provide information beyond that derived from widely used clinical variables has been modest. It is difficult to fully assess the clinical utility of PRSs from these studies because they have used different sets of genetic markers, many of which were derived from early GWASs, and different analytical techniques. For these reasons, it is also difficult to compare PRS utility across ancestry groups, though studies across diverse ancestry groups have often provided qualitatively similar results [13–18]. In the present study, we analyze utility of type 2 diabetes PRSs to predict incident diabetes in African Americans (AfrAm) and European Americans (EurAM) in publicly available data from well-characterized cohorts obtained from the database of genotype and phenotype (dbGAP) [21]. We evaluate several PRSs, including those derived from multi-ancestry GWASs, and we systematically assess several measures of predictive utility.

## Methods

### Participants and measures

Phenotypic and genotypic data were obtained from dbGAP for longitudinal cohorts that have been well-characterized for diabetes and related risk factors. All participants in these studies provided informed consent. For the present analyses, participants were classified as EurAm or AfrAm according to race/ethnicity categories, which were available from self-report in the original studies. While there are limitations with the use of self-reported ancestry, which does not always correspond with genetic ancestry [22, 23], the GWAS data were often generated separately according to self-reported ancestry group, and previous studies have demonstrated potential health disparities with use of PRS across self-reported ancestry groups [4, 24]. Data for the present analyses were obtained for the Atherosclerosis Risk in Communities (ARIC) cohort [25] (2926 AfrAm, 9344 EurAm), the Coronary Artery Risk Development in Young Adults (CARDIA) cohort [26] (1177 AfrAm, 1668 EurAm), the Multiethnic Study of Atherosclerosis (MESA) cohort [27] (1611 AfrAm, 2526 EurAm), and the Framingham cohort [28] (8290 EurAm). For the Framingham cohort, we included the Offspring and Third Generation cohorts, but not the original cohort since only a small proportion of the original cohort had genotypic data available.

Fasting glucose measurements were available at each examination in each cohort, but post-load glucose measurements and glycated hemoglobin were not, so diabetes was diagnosed according to a documented clinical diagnosis or a fasting glucose measurement ≥ 7.0 mmol/l (126 mg/dl) [29]. Analyses of diabetes incidence were conducted in individuals who were without diabetes at their first examination, and who had at least one follow-up examination with data on presence of diabetes. Characteristics of individuals included in the analyses are shown in supplemental Table S1. Clinical screening recommendations for type 2 diabetes risk have often included measures of family history, glycemia and obesity [30]. Therefore, we included parental history of diabetes, fasting glucose and body mass index (BMI), along with age and sex, as clinical predictors of diabetes. Some authorities also recommend consideration of serum lipid measurements [31]; therefore, in some analyses we also included serum high density lipoprotein (HDL) cholesterol and triglyceride concentrations. Fasting glucose is widely used as a clinical predictor of diabetes, but, as a potential mediator, its inclusion may result in underestimation of the genetic contribution to diabetes risk. Therefore, we also report results for models that do not include fasting glucose as a predictor. For each cohort, we generated genetic principal components from the correlations among 13,028 SNPs (selected at intervals of ~ 200 kb to reduce linkage disequilibrium) to use as covariates to account for population stratification [32, 33]. We do not adjust for these estimates in primary analyses, since they may not be readily available in clinical situations, but we include them in supplemental analyses to determine whether results may be influenced by residual population stratification. We also calculated PRSs and principal components for the 2,504 individuals in Phase 3 of the 1000 Genomes data [34], regressed the PRS on the first four principal components (which separate the major continental populations) and applied the adjustment for the projected 1000 Genomes data in each of the cohorts. As described previously, this “ad hoc” procedure can be applied to any sample with suitable genetic data without the need to generate study-specific principal components [6].

### Genotypes

GWAS array data were obtained for each study. Imputation was conducted using the Phase 3 1000 Genomes data as the reference panel [34]. Phasing and imputation were conducted separately for each cohort and each ancestry group with BEAGLE (version 5.2, downloaded February 3, 2023) [35, 36]. Genotypic “dosage” scores were retained for analysis if the imputation r^2^ was > 0.5 and the minor allele frequency was > 0.01. Full details of imputation and final marker sets are described in supplemental methods and Supplemental Table S2.

### Polygenic scores

We evaluated several polygenic scores for type 2 diabetes that have been recently derived from meta-analyses of large GWASs by a variety of methods. The goal was not to systematically evaluate all PRSs proposed, of which there are a large number, nor was the goal to develop new PRSs. Instead, we aimed to evaluate a representative set of PRSs that have been widely used, including scores derived from genome-wide significant variants by pruning and thresholding, such as PGS003729 [3], PGS003730 [4], and a score from Suzuki et al., (no polygenic score catalog number) [5]. We also evaluated a PRS which used the same variants as PGS003730 but with population-specific weights (PGS003735 and PGS003733, for individuals of African and European ancestry, respectively). In addition, we included scores derived by methods that account for linkage disequilibrium among markers, including PGS000014 and PGS001818 (each derived by the LDPRED program) [9, 37], PGS002771 (derived by the PRSice program) [38], and PGS002308 (derived by the PRSCx program) [6]. PRSCx is a Bayesian method that estimates effect sizes across different ancestry groups, accounting for linkage disequilibrium in each group, and these ancestry-specific estimates are then summarized to produce an overall PRS [39]. Characteristics of the different PRSs evaluated are shown in Table 1 with further cohort-specific details in Supplemental Table S3.

**Table 1:** Type 2 diabetes polygenic scores evaluated

Score	Reference	Ancestry	Method	# Variants	Effective *N*
PGS003729	Mahajan et al., 2018 [3]	European	P&T	393	136,013
PGS003730	Mahajan et al., 2022 [4]	Multiple	P&T	338	312,857
Suzuki, 2024	Suzuki et al., 2024 [5]	Multiple	P&T	1,289	712,109
PGS003735 (AfrAm) PGS003733 (EurAm)	Mahajan et al., 2022 [4]	Multiple	P&T	338	varies
PGS000014	Khera et al., 2018 [9]	European	LDPRED	6,913,674	44,413
PGS001818	Prive et al., 2022 [37]	European	LDPRED	30,745	35,038
PGS002771	Mars et al., 2022 [38]	European	PRSice	1,090,067	136,013
PGS002308	Ge et al., 2022 [6]	Multiple	PRSCx	1,104,085	238,662

Score is the number from the polygenic score catalog (https://www.pgscatalog.org), except for Suzuki et al., for which there is no number. Ancestry is the group in which the polygenic score was derived. P&T is pruning and thresholding (here based on genome-wide statistical significance), LDPRED, PRSice and PRSCx are the programs by which polygenic scores were derived. Effective N is the effective sample size in the sample in which the polygenic score was derived- calculated as the harmonic mean of the number of cases and number of controls [40]

Polygenic scores were calculated from the variant-specific weights, which are typically logarithms of odds ratios, potentially accounting for linkage disequilibrium, (*w*_*i*_, being the weight for the ith variant) and the number or “dosage” of risk alleles which the individual carries at carries at each variant (*d*_*i*_). The PRS across *m* markers was calculated as $m\sum\limits_{i=1}^m\;w_id_i/\sum\limits_{i=1}^m\;w_i$. This expresses the PRS as the number of risk alleles carried by each individual, averaged accounting for the weights. If *d*_*i*_ was missing (e.g., due to failure of imputation), it was replaced by its expected value based on the frequency of the risk allele for that variant in the 1000 Genomes group corresponding to the individual’s ancestry group (calculated under the assumption of Hardy-Weinberg proportions). This allows for comparable scaling of the PRS across different cohorts and ancestral groups.

### Diabetes incidence

Statistical analyses were conducted using programs from the SAS Institute (version 9.4, Cary, NC). Person-time for calculation of incidence of diabetes was calculated from the first examination in each cohort until the occurrence of diabetes or until the last examination without diabetes, whichever came first. Incidence rates were calculated for groups of the PRS, with adjustment for age and sex, by Poisson regression [41]. Associations of PRS with diabetes incidence were assessed by proportional hazards regression with adjustment for age, sex, BMI, fasting glucose and parental history of diabetes [42]. The standardized hazard ratio (HR), expressed per standard deviation of the PRS in each cohort and ancestry group, was taken as a measure of strength of association. We also calculated the incremental pseudo-R^2^, a measure of explained randomness for survival models, associated with adding the PRS to the model, with the modification proposed by Royston [43, 44]. Scores with higher standardized HRs and higher pseudo-R^2^ values were considered better performing PRSs. Since standard deviations may vary across ancestry groups, we also report hazard ratios for a fixed difference in PRS (e.g., per 5000 risk alleles) to facilitate comparisons across ancestry groups.

### Discrimination and reclassification

We calculated the C statistic as a measure of the ability of a model to discriminate between those who developed diabetes and those who did not [45]. The C statistic can vary between 0.5 (equal to chance) and 1.0 (perfect discrimination). The change in the C statistic (ΔC) between a model with covariates only and one that also included the PRS was taken as a measure of the PRS’s discriminative ability. C-statistics and their standard errors were calculated as described by Pencina and D’Agostino, while the statistical significance of ΔC was calculated following DeLong et al. [46, 47]. While ΔC is widely used to assess the additional discriminative capability of a model, it depends on C of the baseline model and does not fully reflect the ability of the biomarker to reclassify individual risk. We additionally calculated the continuous net reclassification information (NRI) associated with adding the PRS to the model [48]. The NRI reflects the ability of the marker to reclassify individual risk accurately (events to higher risk, non-events to lower risk); it can range from − 2 to 2, and it provides complementary information to the ΔC [49]. NRI was calculated using methods developed for survival analysis [50]. For comparability across cohorts, NRI was calculated at a follow-up time of 9 years.

### Decision curve analyses

We used decision curve analysis to assess potential clinical utility of adding the PRS to clinical covariates [51]. Decision curve analysis is often used by epidemiologists to model the consequences of clinical decisions in terms of the relative benefit of detecting true positives versus the cost of false positives. It operates under the assumption that if the chance of an event (in this case developing diabetes) exceeds a certain threshold probability (p_t_) then the clinical decision would be made to implement an intervention. The choice of p_t_ implicitly sets the relative costs and benefits and the net benefit is calculated accordingly across a range of p_t_ values. We calculated the standardized net benefit (net benefit divided by its maximum possible value [sNB]) for each cohort across a range of p_t_ values using an extension of decision curve analysis developed for survival data [52, 53]. For context, we also calculate sNB for applying the intervention to all individuals, without any selection of higher risk individuals. The area under the decision curve (AUDC), calculated by the trapezoidal rule, was taken as a summary measure of potential clinical utility across all p_t_ values. For these calculations we only considered positive values of sNB (i.e., negative values were set to 0) and only included values where the proportion of individuals exceeding p_t_ exceeded 0.01 (since sNB is poorly estimated at lower values). The relative improvement in AUDC (ΔAUDC) from adding the PRS was calculated from comparing the logarithm of the AUDC for the model including the PRS to that for covariates only.

### Summary and meta-analyses

Summary measures of the parameters of interest (e.g.. HR, ΔC, NRI, decision curve parameters) were calculated across cohorts using inverse-variance fixed effects meta-analysis methods [54]. Standard errors for the NRI and decision curve analysis parameters used in these analyses were calculated by a bootstrap method [55]. Heterogeneity was assessed using Cochran’s Q and the I^2^ statistic [56]. Fixed effects meta-regression was used to examine the extent to which parameters of interest associated with cohort characteristics, such as mean age [57]. Some of these cohorts were included in the GWAS meta-analyses from which the PRSs were derived. We also used meta-regression to adjust for this to account for potential overfitting due to inclusion of cohorts used in the PRS derivation. Local nonparametric regression techniques were incorporated into the meta-regression to produce smoothing of decision curves (using PROC LOESS in SAS).

## Results

### Comparisons of different polygenic scores

**Fig. 1:**
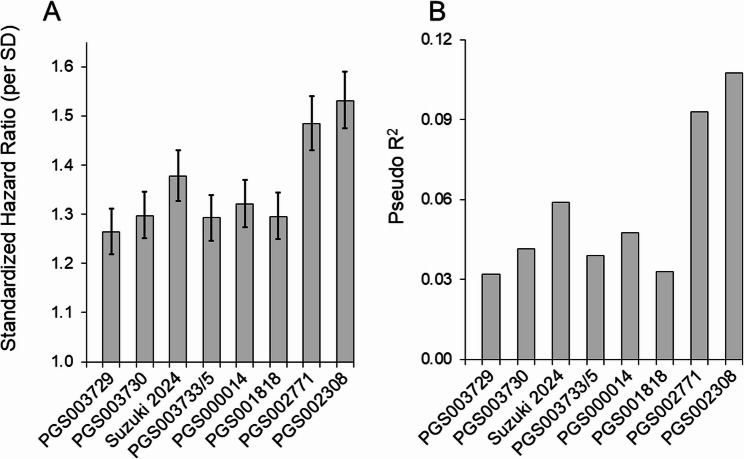
Strength of association for each of the eight PRSs evaluated. **A** Standardized hazard ratio, expressed per SD of the PRS in each cohort and ancestry group. **B** Pseudo-R^2^, a measure of randomness explained. See Table 1 for identity of PRSs

Standardized hazard ratios, with adjustment for age, sex, BMI, parental history of diabetes and baseline fasting glucose, and summarized across all cohorts, are shown in Fig. 1A, for each of the eight PRSs evaluated. All PRSs were strongly and significantly associated with incident diabetes, with standardized HRs ranging from 1.27 for PGS003729 to 1.54 for PGS002308; p-values ranged from 3.0 × 10^− 35^ to 3.8 × 10^− 110^. Pseudo-R^2^ values are shown for each of the PRSs in Fig. 1B; once again PGS002308 had the strongest association with incident diabetes by this measure. Detailed results for PGS002308, which was derived from a multi-ancestry GWAS, and which consists of 1.2 M variants [6], are presented for the rest of this report, as representative of the optimal available PRS. Results for the other PRSs are summarized in supplemental tables S4-S10.

### Diabetes incidence and distribution of PRS

**Fig. 2:**
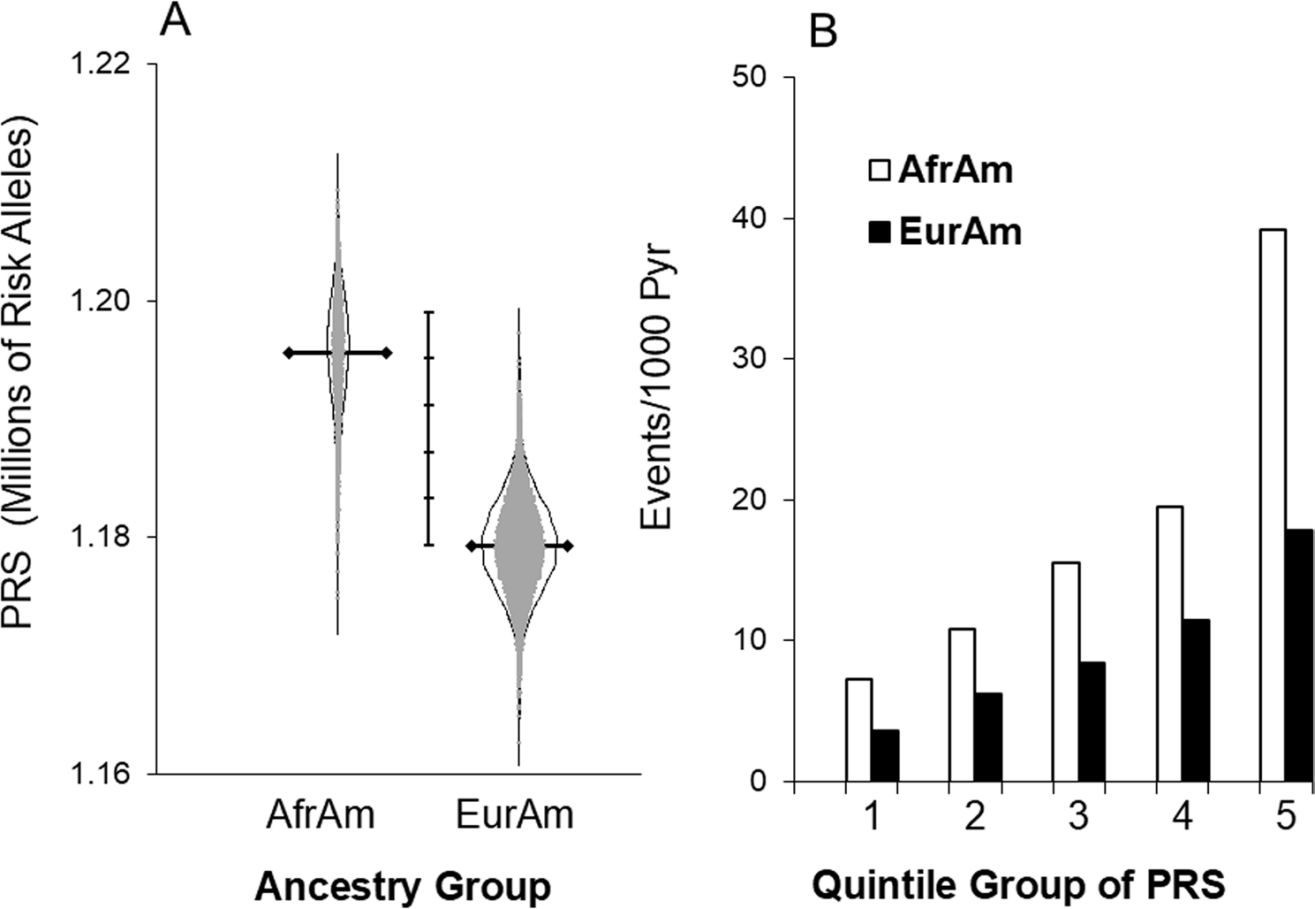
**A** “Bean” plots of distribution of PGS002308 by ancestry group. Gray horizontal lines represent the number of individuals at each level of the PRS; the thick black horizonal lines represent the mean values and the thin curved lines represent the density function. Ticks on the ruled scale represent SD units in the pooled sample across ancestry groups; the pooled SD, calculated as {[((N_A_-1)*SD_A_^2^)+((N_E_-1)*SD_E_^2^)]/(N_A_+N_E_-2)}^½^, where A and E represent AfrAm and EurAm, is 3934 risk alleles. **B** Age-sex adjusted incidence rates of diabetes by cohort specific quintile groups of the PGS002308 (PRS)

There were 21,006 individuals included in the analyses across all cohorts (3,661 AfrAm and 17,345 EurAm). The distribution of PGS002308 in each ancestry group is shown in Fig. 2A. In general scores were higher in AfrAm (mean ± SD= 1.1956 ± 0.0046 M risk alleles) than in EurAm (1.1793 ± 0.0038 M risk alleles); the difference in means was greater than 4 pooled standard deviations. Age-sex adjusted incidence rates of diabetes in ancestry-specific quintile groups of the PRS are shown for each ancestry group in Fig. 2B. Regardless of ancestry, diabetes incidence was higher in higher quintile groups of the PRS than in lower groups. Diabetes incidence rates were higher in AfrAm than in EurAm (incidence rate ratio 1.93, 95% confidence interval [CI] 1.77–2.11). This difference is not as high as might be expected based on the differences in PRS, suggesting that the population differences in PRS are at least partially due to biases in its derivation.

### Measures of utility for PRS and clinical variables

Measures of association, discrimination and reclassification for PGS002308 with incident diabetes are shown in Table 2. Measures for selected clinical variables are shown as well. With adjustment for age, sex, parental history of diabetes, BMI and fasting glucose, PRS was strongly associated with incident diabetes. The overall HR was 1.72 per 5000 risk alleles; the HR was 1.65 in AfrAm and 1.75 in EurAm with little evidence of heterogeneity between ancestry groups. The PRS significantly improved the ability to discriminate between those who develop diabetes and those who do not; compared with a model containing age, sex, parental history, fasting glucose and BMI, ΔC was 0.010 overall, 0.014 in AfrAm and 0.010 in EurAm, again with little evidence of heterogeneity between ancestry groups. PRS also provided significant utility for reclassification of diabetes risk with an NRI of 0.342 overall, 0.379 in AfrAm and 0.326 in EurAm with little evidence for heterogeneity. Among clinical variables, fasting glucose tended to provide the strongest prediction with an NRI overall of 0.807. The effects of the PRS were somewhat weaker than those for BMI in EurAm and somewhat stronger in AfrAm; for example, the NRI for BMI was 0.238 in AfrAm and 0.458 in EurAm. Effects of BMI were significantly weaker in AfrAm than in EurAm by all measures.

**Table 2:** Hazard ratios, improvement in C-statistic (ΔC), Net Reclassification Information (NRI) and Improvement in Area Under Decision Curve (ΔAUDC) for PGS002308 (PRS) and selected clinical predictors of diabetes incidence

Hazard Ratios	AfrAm		EurAm		Combined AfrAm+EurAm			
	Est. (95% CI)	*P*-value	Est. (95% CI)	*P*-value	Est. (95% CI)	*P*-value	I^2^ (%)	*P*_het_
PRS (per 5000 risk alles)*	1.65 (1.50–1.81)	4.0 × 10^− 25^	1.75 (1.66–1.85)	4.4 × 10^− 87^	1.72 (1.64–1.81)	3.8 × 10^− 110^	18	0.270
BMI (per kg/m^2^)	1.05 (1.03–1.06)	2.5 × 10^− 14^	1.10 (1.09–1.11)	6.1 × 10^− 124^	1.08 (1.07–1.09)	2.5 × 10^− 126^	98	6.6 × 10^− 12^
Fasting Glucose (per mM)	3.86 (3.37–4.43)	1.9 × 10^− 82^	4.05 (3.72–4.40)	2.8 × 10^− 236^	4.00 (3.72–4.30)	< 10^− 300^	0	0.58
ΔC								
PRS	0.014 (0.007–0.022)	3.3 × 10^− 4^	0.010 (0.007–0.012)	5.4 × 10^− 11^	0.010 (0.007–0.012)	1.5 × 10^− 13^	23	0.253
BMI	0.006 (0.001–0.011)	0.017	0.020 (0.016–0.025)	3.0 × 10^− 21^	0.014 (0.011–0.018)	1.7 × 10^− 18^	95	2.0 × 10^− 5^
Fasting Glucose	0.042 (0.029–0.054)	7.9 × 10^− 11^	0.016 (0.011–0.020)	3.0 × 10^− 15^	0.018 (0.014–0.022)	2.6 × 10^− 21^	93	1.2 × 10^− 4^
NRI								
PRS	0.379 (0.258–0.499)	7.0 × 10^− 10^	0.326 (0.249–0.404)	2.3 × 10^− 16^	0.342 (0.277–0.407)	1.3 × 10^− 24^	0	0.474
BMI	0.238 (0.115–0.362)	1.6 × 10^− 4^	0.458 (0.384–0.531)	1.9 × 10^− 34^	0.401 (0.338–0.464)	1.3 × 10^− 35^	89	2.8 × 10^− 3^
Fasting Glucose	0.741 (0.631-0.800)	3.2 × 10^− 40^	0.831 (0.766–0.866)	5.1 × 10^− 140^	0.807 (0.752–0.837)	6.4 × 10^− 178^	48	0.165
ΔAUDC (%)								
PRS	8.7 (3.8–13.9)	4.5 × 10^− 4^	5.6 (2.0-9.3)	2.3 × 10^− 3^	6.8 (3.8–9.9)	7.5 × 10^− 6^	0	0.322
BMI	2.5 (-0.4-5.4)	0.092	10.8 (6.8–15.0)	5.6 × 10^− 8^	7.8 (5.1–10.6)	4.8 × 10^− 9^	91	9.8 × 10^− 4^
Fasting Glucose	47.2 (34.3–61.5)	2.1 × 10^− 16^	79.1 (66.9–92.1)	2.1 × 10^− 59^	76.7 (64.6–89.7)	7.7 × 10^− 56^	91	9.5 × 10^− 4^

*Est*. is estimate, *CI* are confidence intervals; *P*-values are for the null hypothesis of no association with the polygenic risk score (HR = 1, ΔC = 0, NRI = 0, ΔAUDC = 0); I^2^ is the proportion of variance in the parameter explained by ancestry group (an estimate of heterogeneity); P_het_ is for the null hypothesis that estimates in AfrAm and EurAm are equal. Alles stands for alleles. Models involve adjustment for age, sex, parental history of diabetes, body mass index and fasting glucose

*A difference of 5000 risk alleles corresponds to 1.27 pooled SD units (see Fig. 2)

### Decision curve analysis

**Fig. 3:**
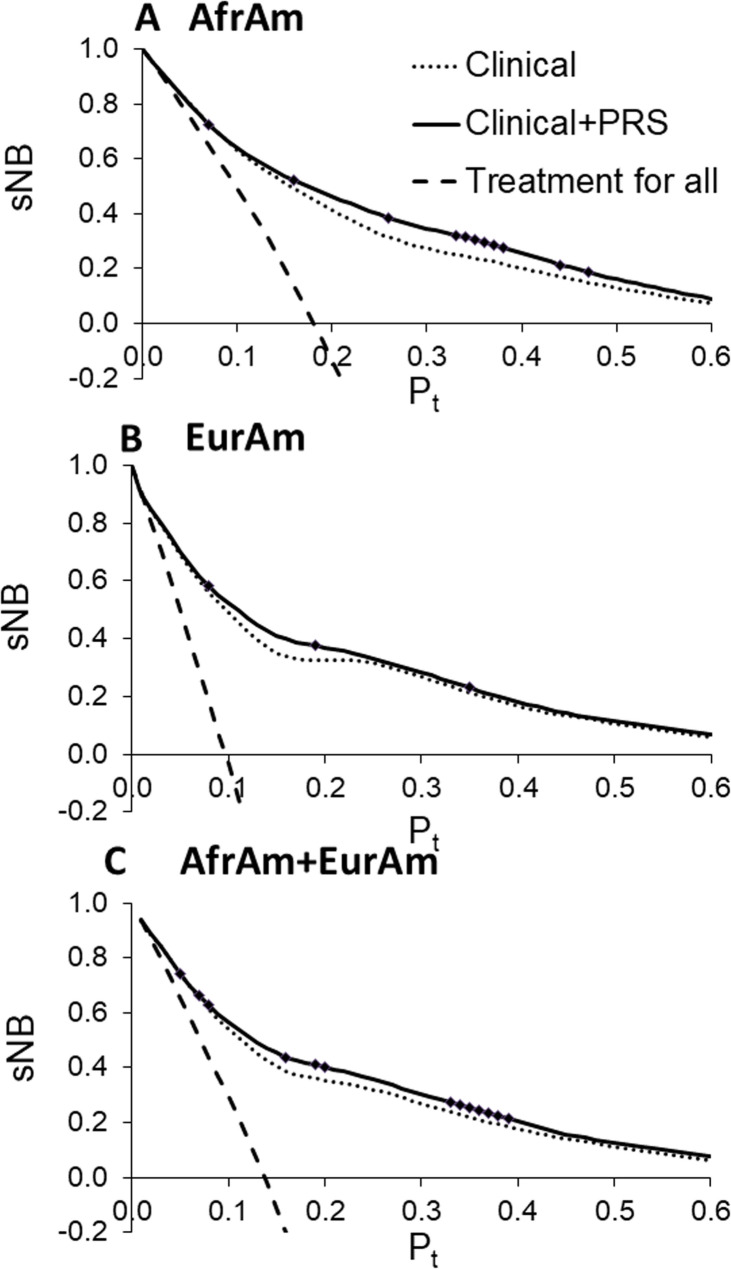
Decision curves for prediction of diabetes for models using clinical variables alone, clinical variables and the PRS, and for a model with treatment for all (without selection of high-risk individuals). sNB is the standardized net benefit, and p_t_ is the threshold probability above which individuals are selected for intervention. At p_t_=0 there are no costs for false positives and sNB = 1 for all curves; at higher p_t_ costs of false positives are higher and strategies that more accurately predict diabetes associate with higher benefit. Points where the PRS provides significantly (*p* < 0.05) greater benefit than clinical variables alone are shown with diamonds. **A** curves for AfrAm, (**B**) curves for EurAm, (**C**) curves for combined sample

Decision curves for both ancestry groups and for the combined sample are shown in Fig. 3. The inclusion of PGS002308 provided significant improvement in the area under the decision curve, and this indicates a potential improvement in clinical benefit with the use of the PRS across all p_t_ values; ΔAUDC was 6.8% overall, 8.7% in AfrAm and 5.6% in EurAm with little indication of heterogeneity between ancestry groups. In general, the relative improvement in standardized net benefit from adding the PRS was greater with more stringent intervention thresholds. For example, among all participants sNB improved by 4.3% at p_t_=0.10, by 10.6% at p_t_=0.20 and by 9.2% at p_t_=0.30; the corresponding values were 1.6%, 9.7% and 15.7% in AfrAm, and 6.2%, 10.2% and 5.4% in EurAm.

### Inclusion of additional covariates

Analyses including HDL cholesterol and triglycerides as additional covariates are shown in Table 3. Inclusion of serum lipids made little difference in the effects associated with PGS002308. Measures of utility for the PRS were somewhat stronger than those for serum lipids. The overall NRI for the PRS was 0.328, while for serum lipids it was 0.257; the corresponding values were 0.364 and 0.208 in AfrAm and 0.314 and 0.277 in EurAm. When fasting glucose was not included as a covariate, the predictive properties associated with PRS were stronger (Supplemental Table S11). To control for potential residual population stratification, we also performed analyses with adjustment for the first four genetic principal components generated in each cohort (Supplemental Table S12). Overall and in EurAm, these results differed little from the main analyses, while the effects of the PRS with this adjustment were slightly stronger in AfrAm. As an alternative approach we also conducted analyses with inclusion of the first four principal components derived from the 1000 Genomes samples projected into each cohort, and we obtained similar results (Supplemental Table S13). These analyses suggest that there is little confounding by population stratification. With the ad hoc approach of adjusting the PRS for principal components projected from the 1000 Genomes samples by the coefficients observed in these same samples, there was little difference in the PRS between ancestry groups (Supplemental Figure S1).

**Table 3:** Hazard ratios, improvement in C-statistic (ΔC), Net Reclassification Information (NRI) and Improvement in Area Under Decision Curve (ΔAUDC) for PGS002308 (PRS) [13] and selected clinical predictors of diabetes incidence with additional adjustment for lipid levels [HDL Cholesterol and Triglycerides]

Hazard Ratios	AfrAm		EurAm		Combined AfrAm+EurAm			
	Est. (95% CI)	*P*-value	Est. (95% CI)	*P*-value	Est. (95% CI)	*P*-value	I^2^ (%)	*P*_het_
PRS (per 5000 risk alles)*	1.63 (1.48–1.79)	5.5 × 10^− 24^	1.73 (1.64–1.83)	1.4 × 10^− 82^	1.70 (1.62–1.79)	1.6 × 10^− 104^	18	0.269
BMI (per kg/m^2^)	1.04 (1.03–1.05)	4.2 × 10^− 12^	1.08 (1.07–1.09)	5.9 × 10^− 81^	1.07 (1.06–1.08)	1.3 × 10^− 85^	96	2.4 × 10^− 7^
Fasting Glucose (per mM)	3.69 (3.20–4.24)	1.6 × 10^− 74^	3.89 (3.58–4.23)	1.0 × 10^− 221^	3.83 (3.57–4.12)	3.9 × 10^− 294^	0	0.520
HDL Cholesterol (per mM)	0.66 (0.52–0.82)	2.3 × 10^− 4^	0.56 (0.48–0.64)	8.0 × 10^− 16^	0.58 (0.52–0.66)	1.7 × 10^− 18^	29	0.234
Triglycerides (per mM)	1.11 (1.04–1.18)	9.4 × 10^− 4^	1.15 (1.11–1.19)	8.0 × 10^− 15^	1.14 (1.11–1.17)	4.6 × 10^− 17^	0	0.376
ΔC								
PRS	0.012 (0.005–0.020)	1.0 × 10^− 3^	0.008 (0.006–0.011)	1.2 × 10^− 9^	0.009 (0.006–0.011)	8.6 × 10^− 12^	10	0.293
BMI	0.004 (-0.001-0.007)	0.092	0.011 (0.008–0.014)	1.2 × 10^− 13^	0.009 (0.006–0.011)	2.7 × 10^− 12^	89	2.8 × 10^− 3^
Fasting Glucose	0.038 (0.026–0.050)	5.0 × 10^− 10^	0.020 (0.015–0.024)	3.3 × 10^− 18^	0.022 (0.018–0.026)	5.0 × 10^− 25^	87	5.6 × 10^− 3^
Lipids	0.006 (0.001–0.011)	0.010	0.007 (0.004–0.009)	1.2 × 10^− 7^	0.007 (0.004–0.009)	4.1 × 10^− 9^	0	0.877
NRI								
PRS	0.364 (0.243–0.484)	3.2 × 10^− 9^	0.314 (0.237–0.391)	1.3 × 10^− 15^	0.328 (0.263–0.393)	3.3 × 10^− 23^	0	0.489
BMI	0.267 (0.144–0.391)	2.3 × 10^− 5^	0.471 (0.398–0.554)	4.8 × 10^− 36^	0.418 (0.355–0.481)	2.8 × 10^− 38^	87	5.5 × 10^− 3^
Fasting Glucose	0.707 (0.593–0.821)	8.5 × 10^− 34^	0.804 (0.739–0.868)	2.8 × 10^− 130^	0.780 (0.724–0.837)	9.1 × 10^− 162^	52	0.149
Lipids	0.208 (0.080–0.337)	1.5 × 10^− 3^	0.277 (0.195–0.360)	4.1 × 10^− 11^	0.257 (0.188–0.327)	3.5 × 10^− 13^	0	0.376
ΔAUDC (%)								
PRS	8.9 (4.0–14.0)	2.7 × 10^− 4^	5.2 (2.2–8.3)	6.5 × 10^− 4^	6.7 (4.0-9.4)	4.2 × 10^− 7^	36	0.211
BMI	1.5 (-1.0-4.1)	0.232	7.5 (4.3–10.8)	2.4 × 10^− 6^	5.5 (3.3–7.7)	8.2 × 10^− 7^	88	4.0 × 10^− 3^
Fasting Glucose	40.0 (27.8–53.3)	4.0 × 10^− 13^	70.5 (59.1–82.8)	3.6 × 10^− 51^	65.3 (54.8–76.6)	2.2 × 10^− 50^	91	7.1 × 10^− 4^
Lipids	1.3 (-1.0-3.6)	0.260	4.3 (1.7–6.9)	1.2 × 10^− 3^	3.2 (1.3–5.1)	9.3 × 10^− 4^	64	0.094

*Est*. is estimate, *CI* are confidence intervals; *P*-values are for the null hypothesis of no association with the polygenic risk score (HR = 1, ΔC = 0, NRI = 0, ΔAUDC = 0); I^2^ is the proportion of variance in the parameter explained by ancestry group (an estimate of heterogeneity); P_het_ is for the null hypothesis that estimates in AfrAm and EurAm are equal. Alles stands for alleles. Models involve adjustment for age, sex, parental history of diabetes, body mass index, fasting glucose, HDL cholesterol and triglyceride levels

^*^A difference of 5000 risk alleles corresponds to 1.27 pooled SD units (see Fig. 2)

### Effects of “Overfitting”

Some cohorts from the present analyses were included in the meta-analyses from which the PRSs were derived; for PGS002308, for example, all AfrAm cohorts and the Framingham and MESA EurAm cohorts were included in the derivation GWAS. This could result in overestimation of the effects of the PRS. To account for this possibility, we used meta-regression to adjust for whether a cohort was included in the derivation GWAS. With this adjustment, the ranks of the different PRSs were largely unchanged with respect to standardized hazard ratios and pseudo-R^2^, though effects of most scores were attenuated (Supplemental Figure S2). Results of analyses with adjustment for overfitting for PGS002308 are shown in Table 4. Hazard ratio estimates for the PRS were moderately attenuated, compared with analyses without the adjustment, particularly for AfrAm, and there was significant heterogeneity between ancestry groups. Other parameters of utility (ΔC, NRI, ΔAUDC) were only modestly attenuated and there were no significant differences between ancestry groups.

**Table 4:** Hazard Ratio (HR), improvement in C-statistic (ΔC), Net Reclassification Information (NRI) and Improvement in Area Under Decision Curve(ΔAUDC) for PGS002308 with adjustment for potential “Overfitting” by meta-regression

Parameter	AfrAm		EurAm		Combined AfrAm+EurAm			
	Est. (95% CI)	*P*-value	Est. (95% CI)	*P*-value	Est. (95% CI)	*P*-value	I^2^ (%)	*P*_het_
HR (per 5000 risk alles)*	1.27 (1.09–1.47)	1.7 × 10^− 3^	1.57 (1.47–1.69)	6.9 × 10^− 34^	1.50 (1.41–1.60)	1.3 × 10^− 34^	91	9.7 × 10^− 4^
ΔC	0.015 (0.005–0.025)	3.1 × 10^− 3^	0.010 (0.006–0.013)	2.6 × 10^− 7^	0.010 (0.007–0.014)	4.5 × 10^− 9^	17	0.273
NRI	0.313 (0.119–0.535)	2.7 × 10^− 3^	0.311 (0.219–0.403)	3.3 × 10^− 11^	0.313 (0.229–0.397)	3.4 × 10^− 13^	0	0.899
ΔAUDC (%)	7.0 (2.4–11.8)	2.7 × 10^− 3^	5.6 (1.1–10.3)	0.014	5.9 (1.6–10.4)	7.3 × 10^− 3^	0	0.687

*Est*. is estimate, *CI* are confidence intervals; *P*-values are for the null hypothesis of no association with the polygenic risk score (HR = 1, ΔC = 0, NRI = 0, ΔAUDC = 0); I^2^ is the proportion of variance in the parameter explained by ancestry group (an estimate of heterogeneity); P_het_ is for the null hypothesis that estimates in AfrAm and EurAm are equal. Alles stands for alleles. Models involve adjustment for age, sex, parental history of diabetes, body mass index, fasting glucose, and were adjusted for potential “overfitting” by a meta-regression approach

^*^A difference of 5000 risk alleles corresponds to 1.27 pooled SD units (see Fig. 2)

Decision curves with adjustment for potential “overfitting” are shown in Supplemental Figure S3. The relative improvement in sNB from inclusion of the PRS over that provided by clinical covariates was only modestly attenuated in comparison to models without this adjustment. ΔAUDC across all p_t_ values was 5.9% overall, 7.0% in AfrAm and 5.6% in EurAm. Among all participants, sNB improved by 3.6% at p_t_=0.10, by 5.5% at p_t_=0.20 and by 7.0% at p_t_=0.30.

### Heterogeneity by age

**Fig. 4:**
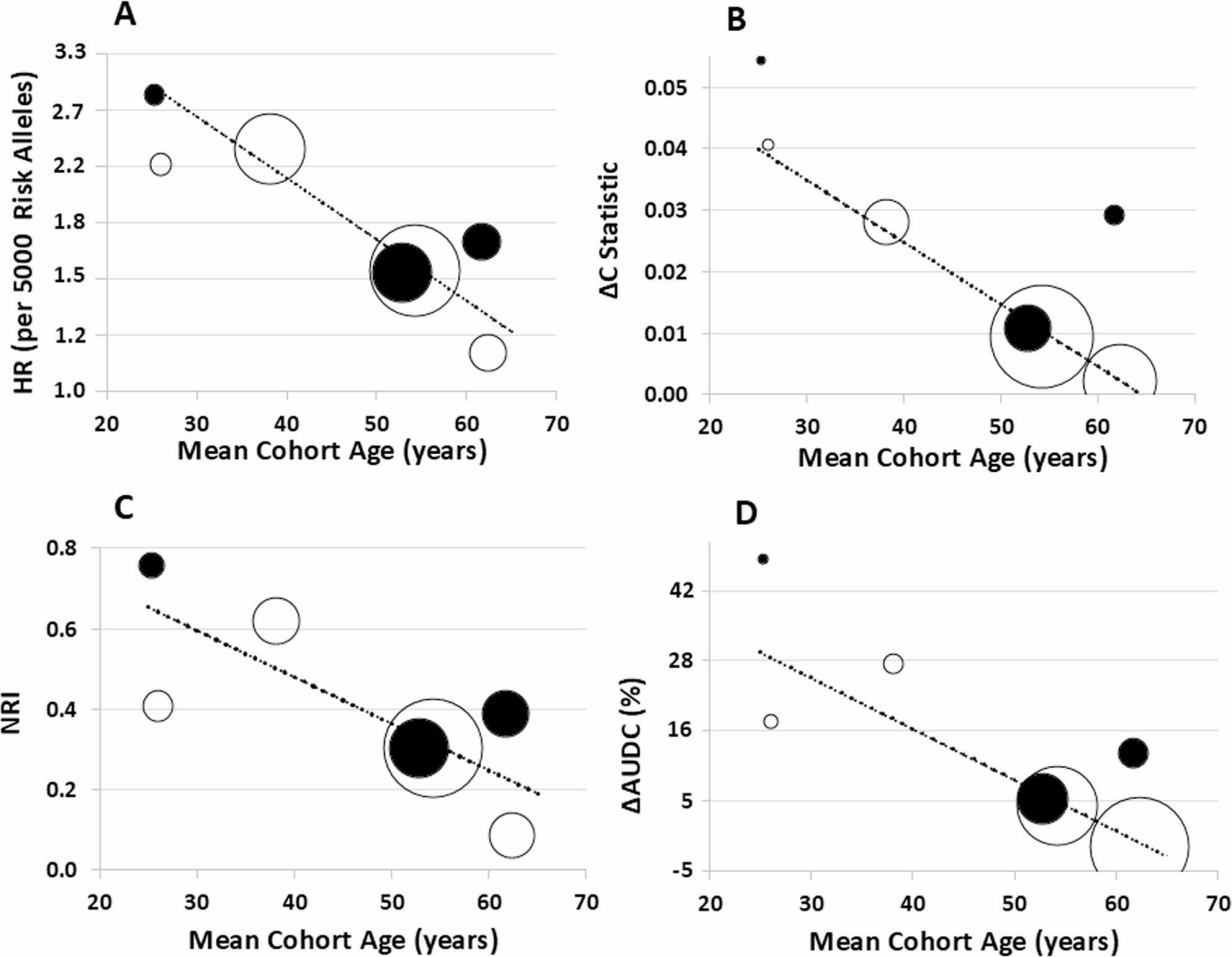
Meta-regression analyses of measures of predictive utility for the PRS against mean cohort age. Dark circles represent AfrAm cohorts, and open circles represent EurAm cohorts. Circle size is proportional to the weight assigned to each cohort inverse of the variance). **A** Analyses of the hazard ratio (HR). **B** Analyses of the change in C statistic (ΔC). **C** Analyses of net reclassification information (NRI). **D** Analyses of change in area under the decision curve (ΔAUDC)

In meta-regression analyses, older mean cohort age was associated with lower values of the HR for the PGS002308, as well as with lower values of ΔC, NRI and ΔAUDC (*P* < 0.001 for each, Fig. 4). The hazard ratio for the PRS was 20% lower per decade of age, while each decade of age was associated with a 0.010 lower ΔC, a 0.116 lower NRI and 7.0% reduction in ΔAUDC.

To further explore potential age differences in utility for prediction of diabetes associated with PGS002308, we conducted analyses with individuals stratified into whether they were younger than age 55 years or at least 55 years old. Age 55 was chosen as the threshold since it is approximately the median in ARIC, the largest cohort. Measures of association and utility were stronger in those under age 55 than in those age ≥ 55 years in both AfrAm and EurAm (Table 5). For example, in the combined sample, the HR per 5000 risk alleles was 1.92 in those under age 55 years and 1.39 in those age ≥ 55; corresponding values were 1.79 and 1.45 in AfrAm and 1.97 and 1.36 in EurAm. Measures of utility for fasting glucose, in contrast, were greater for those age ≥ 55 years.

**Table 5:** Hazard Ratio (HR), improvement in C-statistic (ΔC), Net Reclassification Information (NRI) and Improvement in Area Under Decision Curve(ΔAUDC) for PGS002308 and select clinical predictors with stratification by age

Hazard Ratios	Age < 55 Years							
	AfrAm		EurAm		Combined AfrAm+EurAm			
	Est. (95% CI)	*P*-value	Est. (95% CI)	*P*-value	Est. (95% CI)	*P*-value	I^2^ (%)	*P*_het_
PRS (per 5000 risk alles)*	1.79 (1.59–2.02)	5.1 × 10^− 21^	1.97 (1.84–2.10)	1.8 × 10^− 86^	1.92 (1.81–2.04)	2.3 × 10^− 105^	42	0.191
BMI (per kg/m^2^)	1.05 (1.03–1.06)	1.2 × 10^− 9^	1.11 (1.10–1.12)	9.4 × 10^− 103^	1.09 (1.08–1.10)	5.1 × 10^− 102^	98	2.2 × 10^− 10^
Fasting Glucose (per mM)	3.66 (3.04–4.39)	1.3 × 10^− 43^	3.30 (2.97–3.66)	1.9 × 10^− 111^	3.38 (3.09–3.70)	5.6 × 10^− 153^	0	0.338
ΔC								
PRS	0.020 (0.008–0.031)	6.5 × 10^− 4^	0.018 (0.013–0.023)	5.1 × 10^− 13^	0.018 (0.014–0.023)	1.4 × 10^− 15^	0	0.789
BMI	0.007 (0.001–0.013)	0.029	0.028 (0.022–0.034)	1.7 × 10^− 19^	0.017 (0.013–0.022)	1.7 × 10^− 15^	97	1.7 × 10^− 6^
Fasting Glucose	0.032 (0.018–0.047)	5.8 × 10^− 6^	0.011 (0.007–0.015)	2.0 × 10^− 7^	0.013 (0.009–0.017)	3.7 × 10^− 10^	88	3.8 × 10^− 3^
NRI								
PRS	0.490 (0.322–0.658)	1.0 × 10^− 8^	0.446 (0.346–0.546)	2.1 × 10^− 18^	0.457 (0.371–0.543)	1.5 × 10^− 25^	0	0.657
BMI	0.265 (0.097–0.432)	2.0 × 10^− 3^	0.503 (0.406-0.600)	2.1 × 10^− 24^	0.443 (0.360–0.567)	3.1 × 10^− 25^	83	0.016
Fasting Glucose	0.725 (0.579–0.871)	1.7 × 10^− 22^	0.751 (0.683–0.819)	3.1 × 10^− 103^	0.746 (0.685–0.808)	6.5 × 10^− 124^	0	0.750
ΔAUDC (%)								
PRS	12.3 (4.9–20.3)	8.9 × 10^− 4^	12.3 (5.4–20.0)	3.5 × 10^− 4^	12.2 (7.0-17.7)	2.2 × 10^− 6^	0	0.998
BMI	2.3 (-1.4-6.3)	0.233	11.3 (5.0–18.0)	3.4 × 10^− 4^	7.4 (3.7–11.3)	6.7 × 10^− 5^	82	0.018
Fasting Glucose	44.1 (28.4–61.6)	5.0 × 10^− 10^	79.1 (51.5-111.8)	8.8 × 10^− 12^	74.1 (58.0-91.9)	5.3 × 10^− 29^	77	0.036

	**Age ≥ 55 Years**							
	**AfrAm**		**EurAm**		**Combined AfrAm+EurAm**			
**Hazard Ratios**	**Est. (95% CI)**	*P*-value	**Est. (95% CI)**	*P*-value	**Est. (95% CI)**	*P*-value	**I**^**2**^ **(%)**	**P**_**het**_
PRS (per 5000 risk alles)	1.45 (1.25–1.69)	1.5 × 10^− 6^	1.36 (1.23–1.51)	1.4 × 10^− 9^	1.39 (1.28–1.51)	3.9 × 10^− 14^	0	0.497
BMI (per kg/m^2^)	1.05 (1.03–1.07)	1.1 × 10^− 5^	1.09 (1.07–1.11)	6.2 × 10^− 27^	1.07 (1.06–1.08)	2.6 × 10^− 29^	88	3.7 × 10^− 3^
Fasting Glucose (per mM)	4.17 (3.34–5.21)	2.5 × 10^− 36^	6.29 (5.45–7.25)	4.6 × 10^− 140^	5.57 (4.94–6.29)	1.7 × 10^− 172^	89	2.3 × 10^− 3^
ΔC								
PRS	0.010 (-0.002-0.023)	0.108	0.003 (0.000-0.007)	0.055	0.004 (0.001–0.007)	0.023	12	0.285
BMI	0.006 (-0.004-0.015)	0.226	0.012 (0.006–0.019)	1.4 × 10^− 4^	0.010 (0.005–0.016)	1.3 × 10^− 4^	26	0.245
Fasting Glucose	0.102 (0.069–0.135)	1.0 × 10^− 9^	0.097 (0.080–0.114)	1.6 × 10^− 30^	0.098 (0.083–0.113)	1.2 × 10^− 38^	0	0.776
NRI								
PRS	0.267 (0.081–0.452)	4.9 × 10^− 3^	0.166 (0.051–0.282)	4.8 × 10^− 3^	0.194 (0.096–0.293)	1.0 × 10^− 4^	0	0.369
BMI	0.281 (0.103–0.459)	2.0 × 10^− 3^	0.428 (0.316–0.540)	6.0 × 10^− 14^	0.387 (0.292–0.481)	1.2 × 10^− 15^	47	0.171
Fasting Glucose	0.734 (0.552–0.917)	3.0 × 10^− 15^	0.960 (0.869–1.051)	1.8 × 10^− 94^	0.915 (0.833–0.996)	5.2 × 10^− 107^	79	0.030
ΔAUDC (%)								
PRS	6.6 (0.3–13.3)	0.038	2.5 (-0.7-5.8)	0.125	3.4 (0.5–6.4)	0.022	20	0.262
BMI	4.0 (-0.4-8.6)	0.074	10.0 (4.8–15.5)	1.1 × 10^− 4^	8.3 (4.4–12.3)	1.8 × 10^− 5^	66	0.088
Fasting Glucose	51.4 (30.7–75.4)	3.1 × 10^− 8^	99.1 (71.6-130.9)	9.0 × 10^− 20^	80.5 (62.8-100.1)	4.0 × 10^− 29^	85	0.010

*Est*. is estimate, *CI* are confidence intervals; *P*-values are for the null hypothesis of no association with the polygenic risk score (HR = 1, ΔC = 0, NRI = 0, ΔAUDC = 0); I^2^ is the proportion of variance in the parameter explained by ancestry group (an estimate of heterogeneity); P_het_ is for the null hypothesis that estimates in AfrAm and EurAm are equal. Alles stands for alleles. Models involve adjustment for age, sex, parental history of diabetes, body mass index, and fasting glucose

*A difference of 5000 risk alleles corresponds to 1.27 pooled SD units (see Fig. 2)

**Fig. 5:**
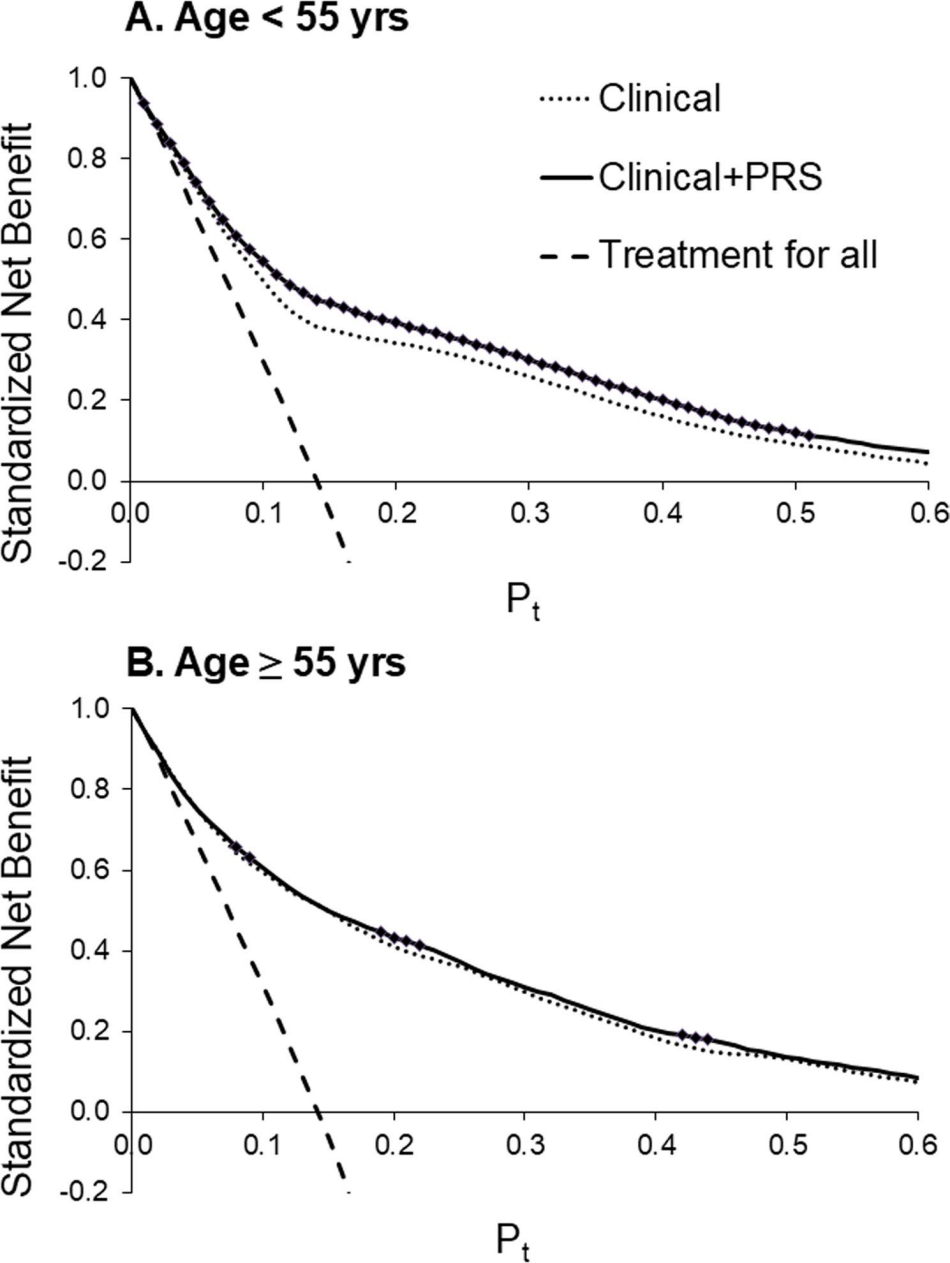
Decision curves for prediction of diabetes for models using clinical variables alone, clinical variables and the PRS, and for a model with treatment for all (without selection of high-risk individuals). sNB is the standardized net benefit, and p_t_ is the threshold probability above which individuals are selected for intervention. At p_t_=0 there are no costs for false positives and sNB = 1 for all curves; at higher p_t_ costs of false positives are higher and strategies that more accurately predict diabetes associate with higher benefit. Points where the PRS provides significantly (*p* < 0.05) greater benefit than clinical variables alone are shown with diamonds. **A** curves for individuals younger than age 55 years (**B**) curves for individuals age ≥ 55 years

Decision curve analysis showed that improvement in relative benefit from adding the PRS to clinical variables was greater in those under age 55 than in those age ≥ 55 years and above (Fig. 5). In the combined group, ΔAUDC was 12.2% in those under age 55 versus 3.4% in those age ≥ 55 years. In those under age 55 years, percent improvement in sNB was 9.5% at P_t_=0.1, 14.4% at P_t_=0.2 and 16.0% at P_t_=0.3; for those age 55 and above corresponding numbers were 2.0%, 5.7% and 4.5%. Results were similar in both AfrAm and EurAm (Supplemental Figure S4).

## Discussion

### PRSs associate with incident diabetes

In recent years, large scale GWASs have facilitated the development of PRSs for many conditions, and there is considerable interest in using these scores in clinical medicine and direct-to-consumer genetic testing. PRSs for type 2 diabetes may be useful for a variety of purposes, including risk stratification and selection of therapies. We evaluated the utility of PRSs for predicting future incidence of diabetes in cohorts of individuals without diabetes initially; this may allow selection of high-risk individuals for preventive interventions. We found that most of the PRSs we evaluated associated strongly with incident diabetes in both AfrAm and EurAm, with adjustment for clinical covariates. PRSs derived from larger GWASs, with larger numbers of markers and from multi-ancestry cohorts tended to perform better, while several PRSs derived from European ancestry populations (PGS003729, PGS000014, PGS002771) showed substantially poorer performance in AfrAm compared with EurAm. PGS002308, which contains 1.2 M variants and which was derived from a large multi-ancestry meta-analysis [6], provided the best performance in the present study. A recent cross-sectional study also showed that PGS002308 performed well across multiple biobank cohorts of diverse ancestries [58]. With larger GWASs composed of more diverse ancestry groups, one would expect better performance from future PRSs.

### Measures of predictive utility

The extent to which PRSs provided additional predictive utility, beyond that provided by clinical factors, was modest to moderate. The increment in the C statistic of ~ 0.01, while highly statistically significant, suggests a modest improvement in the ability of the PRS to discriminate between those who will and will not develop diabetes; the clinical relevance of this is uncertain. The ΔC statistic, however, is strongly dependent on the baseline value of C; with an overall baseline C = 0.81 observed in the present study, ΔC ~ 0.01 suggests a predictor of intermediate strength [49]. Furthermore, ΔC may not fully reflect the ability of the PRS to reclassify individuals according to diabetes risk. The NRI, on the other hand, is a measure of reclassification that is less dependent on strength of the other covariates [49]. The NRIs observed with the PRS in the present analyses of ~ 0.3 are also suggestive of a predictor of intermediate strength and they are comparable to those for other widely used clinical predictors [49]. In fact, the measures of predictive utility observed here for the PRS compare favorably with those for clinical variables often used to assess diabetes risk. While measures of fasting glucose had the best measures of utility, measures for the PRS were of similar magnitude to those for BMI, being slightly lower in EurAm and slightly higher in AfrAm, and they were modestly better than those for serum lipids. Some of the cohorts included in the present analyses were also included in those used to derive the PRSs, and this may have resulted in an overestimation of the utility for the PRS. Our meta-regression analyses suggest that any such overestimation is modest, but this method may lack precision, particularly for the AfrAm cohorts.

### Decision curve analyses

The potential additional clinical utility of PRSs for assessing diabetes risk is probably best captured by the decision curve analyses. The improvement in AUDC of ~ 6% suggests a modest improvement in potential benefit of including the PRS in addition to clinical factors across all p_t_ levels; the improvements are somewhat larger at more stringent p_t_ values. Although these improvements in benefit are modest, they are potentially meaningful on a population basis. It is difficult to assign p_t_ values for current diabetes prevention practices, which vary widely. In the United States, eligibility criteria for the National Diabetes Prevention Program are expansive; 48% of adults meet the criteria for a lifestyle intervention, whereas only 13% meet criteria for enrollment in the original clinical trial demonstrating effectiveness of this intervention [59]. The broader criteria are consistent with a lower p_t_ value, while the more restrictive criteria suggest a higher p_t_, at which the PRS may provide greater benefit. Decision curve analyses are made under the assumption that the benefit (i.e., effectiveness of intervention) does not depend on how high-risk individuals are selected. Analyses of prevention studies in the US and Finland suggest that lifestyle intervention is effective for diabetes prevention regardless of PRS, in some cases even in those at low clinical risk, but further studies are needed [60, 61]. Diabetes can be effectively prevented by lifestyle, pharmacologic and surgical interventions [62], and it is likely that optimal p_t_ values depend on the type of intervention.

### Differences between ancestry groups

PRSs often exhibit large differences among ancestry groups, and these differences may reflect biases due to variant ascertainment for GWAS arrays and varying patterns of linkage disequilibrium, instead of population differences in genetic disease risk [63, 64]. For PGS002308, we observed large differences between AfrAm and EurAm, and these differences were largely eliminated with adjustment for principal components projected from the 1000 Genomes data. Thus, this ad hoc adjustment method may facilitate clinical interpretation of the PRS, making it comparable across different ancestry groups. PRSs often show weaker associations in populations genetically different from those in which they were derived, and associations in populations of African origin are often weak even when the PRS is derived from a multi-ancestry GWAS [63, 64]. Thus, there is concern that clinical use of PRSs may exacerbate health disparities [24]. The present study suggests this may not be the case when using PGS002308 to predict incident diabetes. While, after adjustment for overfitting, the strength of association as measured by the HR was weaker in AfrAm than in EurAm, the other measures of utility (ΔC, NRI, ΔAUDC) did not differ substantially between the groups. The additional clinical benefit of incorporating a PRS does not depend solely on strength of association; it also depends on utility of the clinical predictors and on population risk. The clinical measures, particularly BMI, tended to be weaker predictors in AfrAm than in EurAm. Further work is needed to assess whether PRSs provide comparable estimates of genetic risk in AfrAm and EurAm. Moreover, the present analyses were restricted to EurAm and AfrAm, and additional studies are needed to assess generalizability to other populations.

### Age differences

The present analyses showed age differences in the extent to which the PRS predicts incident diabetes, with measures of predictive utility being stronger in individuals under 55 years than in those age 55 and older. Previous studies have also shown that associations between PRSs and type 2 diabetes, and other chronic diseases, are stronger in younger than in older individuals [18, 58, 65]. These findings suggest that the relative increase in benefit from incorporating the PRS, in addition to clinical predictors, into diabetes risk assessment for preventive purposes is likely to be greater for younger than for older individuals. Nonetheless, utility for older individuals was not negligible, and they are at higher risk for diabetes, so it may still be worthwhile to consider using the PRS in older individuals.

## Conclusions

The present analyses suggest that PRSs provide modest, but potentially meaningful, utility for prediction of diabetes for the purposes of clinical prevention. The relative utility, beyond that provided by standard clinical predictors, is likely greater for younger than for older individuals. Further studies, including cost effectiveness analyses, are required to determine optimal target populations for diabetes prevention efforts; such studies should consider the use of type 2 diabetes polygenic risk scores.

## Supplementary Information

Supplementary Material 1.

## Data Availability

The datasets analyzed during the current study are available in the database of Genotype and Phenotype (dbGAP) repository (https://dbgap.ncbi.nlm.nih.gov/home/). Data obtained from dbGAP were based on the following accession numbers: ARIC- phs000280.v8.p2; CARDIA- phs000285.v3.p2; Framingham Cohort- phs000007.v35.p1; MESA- phs000209.v13.p3.
