# Supplementary material for: Quantifying the utility of type 2 diabetes polygenic risk score for predicting incident diabetes: an analysis of large US-based cohort studies
Source: BMC Med Genomics. 2026 May 12;19:111. doi: 10.1186/s12920-026-02386-7 (PMC13343701; doi:10.1186/s12920-026-02386-7)
Supplement: Supplementary file 1 — Supplementary Material 1. [file 12920_2026_2386_MOESM1_ESM.pdf]

SUPPLEMENTAL TEXT

Phenotypes and Covariates: Phenotypic data from all cohorts were obtained from dbGAP [1]. Diabetes was defined based on a clinical history consistent with the disease or a fasting glucose level ≥7.0 mmol/l (126 mg/dl) [2]. All cohorts measured fasting glucose levels at all/most examinations, but did not routinely measure hemoglobin A1c or perform glucose tolerance tests, so diabetes diagnoses were based on fasting glucose. In the ARIC, Framingham and MESA cohorts, clinical history was based on current treatment with antidiabetic medicines. In the CARDIA cohorts, history was based on self-report of clinician-diagnosed diabetes. Individuals who did not have diabetes at their first examination and who had at least one follow-up examination were included in analyses of diabetes incidence. Follow-up time was calculated as the time to development of diabetes or until last examination without diabetes if the event did not occur. In the ARIC and Framingham cohorts, the date of diabetes diagnosis was taken as the first examination at which diabetes was observed. In the CARDIA and MESA cohorts, information on age at clinical diagnosis was also used where available. For the ARIC, CARDIA and MESA cohorts, parental history of diabetes was obtained at the baseline examination by self-report from the participants. In ARIC, family history for each parent was recorded as present or absent, so two variables, one for each parent, were used to account for parental history. In CARDIA and MESA, parental diabetes was reported as "yes", "no" or "unknown", and four variables were used to represent the corresponding categories for both parents. In the Framingham cohort, family history was not available at baseline, and parental diabetes information was obtained from the last examination conducted in the parent prior to the offspring's baseline examination. Parents were, thus, categorized as having diabetes, not having diabetes or of unknown status, based on these examinations. Information on maternal diabetes was available in 72% of participants, while information on paternal diabetes was available in 67%.

Genotypes and Imputation: Genotypic data from GWAS arrays for each cohort were obtained from dbGAP, in PLINK or text format [1]. Data for the ARIC, CARDIA and MESA cohorts were generated on the Affymetrix Human SNP Array 6.0, while data for Framingham were generated

on the Affymetrix Human SNP Array 5.0 and the Affymetrix GeneChip Human Mapping 50K Array. Autosomal SNPs were selected for analysis if they were included in the 1000 Genomes Phase 3 data with consistent alleles [3], if they were typed in over 90% of individuals in the cohort and if the distribution of genotypes was within Hardy-Weinberg proportions; since p-values depend on sample size, the p-value for determining Hardy-Weinberg varied by cohort. Phasing and imputation were performed, with the 1000 Genomes data (build 37) as a reference, with BEAGLE (https://faculty.washington.edu/browning/beagle/beagle.html version 5.2, downloaded February 3, 2023) [4, 5]. In the Framingham cohort, where parental genotypes were available for a substantial number of participants, the phasing incorporated parental genotypes where available; genotypic information was available on both parents for 24% of participants, and on one parent for 27%. Imputation was conducted using genetic maps constructed by the International HapMap Project [6]. Imputed SNPs with imputation $r^2>0.5$ and minor allele frequency ≥ 0.01 were retained for analysis. Numbers of directly genotyped and successfully imputed SNPs for each cohort are shown in Supplemental Table S2.

Table S1- Characteristics of Individuals Included by Cohort

Cohort	Ancestry	N (Men/Women)	Age Range (yrs)	Mean Age (yrs ± SD)	Mean BMI (kg/m^2 ± SD)	Mean Fasting Glucose (mM ± SD)	Mean Follow-Up (yrs ± SD)	Developed Diabetes- N (%)
ARIC	AfrAm	1612 (613/999)	44-66	52.8 ± 5.7	29.1 ± 5.9	5.46 ± 0.55	11.5 ± 7.8	441 (27.3)
ARIC	EurAm	7569 (3504/4065)	44-66	54.2 ± 5.7	26.7 ± 4.6	5.48 ± 0.50	13.7 ± 8.1	1297 (17.1)
CARDIA	AfrAm	823 (338/485)	20-34	25.3 ± 3.2	25.7 ± 5.8	4.49 ± 0.48	13.5 ± 2.9	63 (7.7)
CARDIA	EurAm	1370 (642/728)	20-32	26.0 ± 2.9	23.7 ± 3.8	4.58 ± 0.44	13.9 ± 2.6	75 (5.5)
Framingham	EurAm	6129 (2851/3278)	20-72	38.1 ± 9.1	26.0 ± 4.8	5.35 ± 0.52	22.0 ± 11.4	735 (12.0)
MESA	AfrAm	1226 (562.664)	45-84	61.7 ± 10.2	29.8 ± 5.8	5.01 ± 0.59	7.3 ± 2.8	184 (15.0)
MESA	EurAm	2277 (1081/1196)	44-84	62.3 ± 10.2	27.5 ± 4.9	4.87 ± 0.55	7.9 ± 2.7	197 (8.7)
Combined	AfrAm	3661 (1513/2148)	20-84	49.6 ± 15.4	28.6 ± 6.1	5.09 ± 0.67	10.5 ± 6.1	688 (18.8)
Combined	EurAm	17345 (8078/9267)	20-84	47.1 ± 13.6	26.3 ± 4.7	5.28 ± 0.58	15.9 ± 10.0	2304 (13.3)

ARIC- Atherosclerosis Risk in Communities; CARDIA- Coronary Artery Risk Development in Young Adults; MESA-Multiethnic Study of Atherosclerosis; AfrAm- African American; EurAm- European American

Table S2: Genotyped and Imputed Variants across Cohorts

Cohort	Ancestry	# Individuals	HW P-value	# GWAS SNPs	# Imputed SNPs
ARIC	AfrAm	2,926	$>1\times10^{-7}$	787,650	14,576,732
ARIC	EurAm	9,344	$>1\times10^{-16}$	786,842	7,999,368
CARDIA	AfrAm	1,177	$>1\times10^{-6}$	801,682	14,447,213
CARDIA	EurAm	1,668	$>1\times10^{-6}$	705,985	7,416,606
Framingham	EurAm	8,290	$>1\times10^{-15}$	490,684	7,563,236
MESA	AfrAm	1,611	$>1\times10^{-6}$	847,716	14,521,365
MESA	EurAm	2,526	$>1\times10^{-6}$	842,732	8,057,081

HW p-value is the p-value for Hardy-Weinberg proportions used to select SNPs for the cohort. # of GWAS SNPs is the number of SNPs passing quality control measures from the GWAS array used for imputation. #of Imputed SNPs is the number of SNPs imputed from the 1000 Genomes Phase 3 reference panel with imputation $r^2>0.5$ and minor allele frequency ≥0.01.

Table S3: Number of SNPs Successfully Imputed for Each Polygenic Score According to Cohort

Score	Ref	# SNPs	ARIC-AfrAm	ARIC-EurAm	CARDIA-AfrAm	CARDIA-EurAm	Fram.-EurAm	MESA-AfrAm	MESA-EurAm
PGS003729	Mahajan et al, 2018 [7]	389	316	342	319	309	325	324	342
PGS003730	Mahajan et al, 2022 [8]	338	302	310	302	283	293	306	310
Suzuki, 2024	Suzuki et al, 2024 [9]	1,289	1,094	1,137	1,095	1,032	1,071	1,109	1,141
PGS003735/ PGS003733	Mahajan et al, 2022 [8]	338	302	310	302	283	293	306	310
PGS000014	Khera et al, 2018 [10]	6,913,685	5,639,004	6,337,963	5,665,238	5,885,936	5,999,320	5,733,708	6,348,910
PGS001818	Privé et al, 2022 [11]	30,775	30,406	30,291	30,337	28,189	29,214	30,487	30,322
PGS002771	Mars et al, 2022 [12]	1,090,967	1,066,825	1,072,521	1,066,794	1,016,147	1,042,824	1,071,112	1,074,608
PGS002308	Ge et al, 2022 [13]	1,258,434	1,223,888	1,104,085	1,224,345	1,045,922	1,076,841	1,228,517	1,108,540

#SNPs is the total number of SNPs in each polygenic score. The number listed for each cohort is the number of SNPs imputed with imputation $r^2>0.5$ and minor allele frequency ≥0.01.

Table S4: Hazard Ratio (HR), Improvement in C-statistic (ΔC), Net Reclassification Information (NRI) and Improvement in Area Under Decision Curve(ΔAUDC) for PGS003729 [7]

	AfrAm		EurAm		Combined AfrAm+EurAm			
**Parameter**	**Est. (95% CI)**	**P-value**	**Est. (95% CI)**	**P-value**	**Est. (95% CI)**	**P-value**	**I^2 (%)**	**P_{het}**
HR (per 10 risk alles)	1.18 (1.06-1.31)	1.7×10^{-3}	1.49 (1.40-1.59)	3.0×10^{-36}	1.40 (1.33-1.48)	3.0×10^{-35}	93	1.5×10^{-4}
ΔC	0.002 (-0.002-0.007)	0.284	0.005 (0.003-0.007)	4.2×10^{-6}	0.005 (0.003-0.006)	4.1×10^{-6}	10	0.291
NRI	0.158 (0.040-0.276)	8.6×10^{-3}	0.224 (0.144-0.303)	3.2×10^{-8}	0.203 (0.137-0.269)	1.4×10^{-9}	0	0.363
ΔAUDC (%)	1.2 (-1.0-3.5)	0.292	2.3 (0.1-4.5)	0.039	2.2 (0.5-3.8)	9.9×10^{-3}	0	0.498

Est. is estimate; CI are confidence intervals; P-values are for the null hypothesis of no association with the polygenic risk score (HR=1, ΔC=0, NRI=0, ΔAUDC=0); I^2 is the proportion of variance in the parameter explained by ancestry group (an estimate of heterogeneity); P_{het} is for the null hypothesis that estimates in AfrAm and EurAm are equal. Alles stands for alleles. Models involve adjustment for age, sex, parental history of diabetes, body mass index and fasting glucose.

Table S5: Hazard Ratio (HR), Improvement in C-statistic (ΔC), Net Reclassification Information (NRI) and Improvement in Area Under Decision Curve(ΔAUDC) for PGS003730 [8]

	AfrAm		EurAm		Combined AfrAm+EurAm			
**Parameter**	**Est. (95% CI)**	**P-value**	**Est. (95% CI)**	**P-value**	**Est. (95% CI)**	**P-value**	**I^2 (%)**	**P_{het}**
HR (per 10 risk alles)	1.20 (1.10-1.31)	5.6×10^{-5}	1.39 (1.33-1.46)	8.8×10^{-44}	1.35 (1.29-1.40)	2.4×10^{-45}	89	2.6×10^{-3}
ΔC	0.001 (-0.002-0.005)	0.421	0.006 (0.004-0.009)	1.7×10^{-8}	0.005 (0.003-0.007)	3.4×10^{-7}	84	0.011
NRI	0.069 (-0.040-0.178)	0.217	0.240 (0.162-0.318)	1.7×10^{-9}	0.182 (0.119-0.245)	1.9×10^{-8}	84	0.012
ΔAUDC (%)	0.6 (-1.6-2.8)	0.593	3.3 (0.2-6.5)	0.038	2.6 (0.4-4.9)	0.019	47	0.168

Est. is estimate; CI are confidence intervals; P-values are for the null hypothesis of no association with the polygenic risk score (HR=1, ΔC=0, NRI=0, ΔAUDC=0); I^2 is the proportion of variance in the parameter explained by ancestry group (an estimate of heterogeneity); P_{het} is for the null hypothesis that estimates in AfrAm and EurAm are equal. Alles stands for alleles. Models involve adjustment for age, sex, parental history of diabetes, body mass index and fasting glucose.

Table S6: Hazard Ratio (HR), Improvement in C-statistic (ΔC), Net Reclassification Information (NRI) and Improvement in Area Under Decision Curve(ΔAUDC) for Suzuki 2024 [9]

	AfrAm		EurAm		Combined AfrAm+EurAm			
**Parameter**	**Est. (95% CI)**	**P-value**	**Est. (95% CI)**	**P-value**	**Est. (95% CI)**	**P-value**	**I^2 (%)**	**P_{het}**
HR (per 20 risk alles)	1.32 (1.19-1.46)	1.1×10^{-7}	1.59 (1.50-1.68)	1.0×10^{-58}	1.52 (1.45-1.60)	1.2×10^{-62}	90	1.5×10^{-3}
ΔC	0.003 (-0.001-0.007)	0.180	0.009 (0.006-0.011)	7.6×10^{-10}	0.007 (0.004-0.009)	5.3×10^{-9}	82	0.018
NRI	0.194 (0.075-0.314)	1.4×10^{-3}	0.271 (0.197-0.344)	5.5×10^{-13}	0.250 (0.187-0.312)	5.6×10^{-15}	12	0.285
ΔAUDC (%)	1.3 (-1.0-3.8)	0.274	4.9 (1.5-8.3)	3.9×10^{-3}	3.8 (1.5-6.2)	1.4×10^{-3}	64	0.095

Est. is estimate; CI are confidence intervals; P-values are for the null hypothesis of no association with the polygenic risk score (HR=1, ΔC=0, NRI=0, AUDC=0); I^2 is the proportion of variance in the parameter explained by ancestry group (an estimate of heterogeneity); P_{het} is for the null hypothesis that estimates in AfrAm and EurAm are equal. Alles stands for alleles. Models involve adjustment for age, sex, parental history of diabetes, body mass index and fasting glucose.

Table S7: Hazard Ratio (HR), Improvement in C-statistic (ΔC), Net Reclassification Information (NRI) and Improvement in Area Under Decision Curve(ΔAUDC) for PGS003733 (EurAm) and PGS003735 (AfrAm) [8]

	AfrAm		EurAm		Combined AfrAm+EurAm			
**Parameter**	**Est. (95% CI)**	**P-value**	**Est. (95% CI)**	**P-value**	**Est. (95% CI)**	**P-value**	**I^2 (%)**	**P_{het}**
HR (per SD)	1.13 (1.05-1.22)	1.4×10^{-3}	1.34 (1.29-1.49)	1.3×10^{-44}	1.29 (1.25-1.34)	1.1×10^{-43}	93	1.3×10^{-4}
ΔC	0.002 (-0.002-0.006)	0.292	0.007 (0.004-0.009)	3.1×10^{-8}	0.005 (0.003-0.007)	1.6×10^{-7}	77	0.038
NRI	0.083 (-0.031-0.198)	0.152	0.246 (0.167-0.324)	9.1×10^{-10}	0.193 (0.129-0.258)	4.7×10^{-9}	81	0.022
ΔAUDC (%)	0.4 (-1.4-2.2)	0.675	3.4 (0.4-6.6)	0.027	2.7 (0.6-4.8)	0.014	65	0.091

Analysis uses population specific weights for variants in PGS003730. Est. is estimate; CI are confidence intervals; P-values are for the null hypothesis of no association with the polygenic risk score (HR=1, ΔC=0, NRI=0, ΔAUDC=0); I^2 is the proportion of variance in the parameter explained by ancestry group (an estimate of heterogeneity); P_{het} is for the null hypothesis that estimates in AfrAm and EurAm are equal. Alles stands for alleles. Models involve adjustment for age, sex, parental history of diabetes, body mass index and fasting glucose.

Table S8: Hazard Ratio (HR), Improvement in C-statistic (ΔC), Net Reclassification Information (NRI) and Improvement in Area Under Decision Curve(ΔAUDC) for PGS000014 [10]

	AfrAm		EurAm		Combined AfrAm+EurAm			
**Parameter**	**Est. (95% CI)**	**P-value**	**Est. (95% CI)**	**P-value**	**Est. (95% CI)**	**P-value**	**I^2 (%)**	**P_{het}**
HR (per 20,000 risk alles)	1.10 (0.99-1.22)	0.091	1.60 (1.51-1.68)	6.3×10^{-64}	1.48 (1.41-1.55)	1.3×10^{-56}	97	1.5×10^{-9}
ΔC	0.000 (-0.003-0.004)	0.838	0.004 (0.002-0.006)	3.9×10^{-5}	0.003 (0.001-0.005)	8.6×10^{-4}	75	0.044
NRI	0.102 (-0.014-0.217	0.086	0.159 (0.085-0.232)	2.4×10^{-5}	0.142 (0.080-0.205)	7.4×10^{-6}	0	0.414
ΔAUDC (%)	0.2 (-1.3-1.7)	0.841	2.6 (0.3-4.9)	0.028	2.0 (0.4-3.7)	0.015	66	0.084

Est. is estimate; CI are confidence intervals; P-values are for the null hypothesis of no association with the polygenic risk score (HR=1, ΔC=0, NRI=0, ΔAUDC=0); I^2 is the proportion of variance in the parameter explained by ancestry group (an estimate of heterogeneity); P_{het} is for the null hypothesis that estimates in AfrAm and EurAm are equal. Alles stands for alleles. Models involve adjustment for age, sex, parental history of diabetes, body mass index and fasting glucose.

Table S9: Hazard Ratio (HR), Improvement in C-statistic (ΔC), Net Reclassification Information (NRI) and Improvement in Area Under Decision Curve(ΔAUDC) for PGS001818 [11]

	AfrAm		EurAm		Combined AfrAm+EurAm			
**Parameter**	**Est. (95% CI)**	**P-value**	**Est. (95% CI)**	**P-value**	**Est. (95% CI)**	**P-value**	**I^2 (%)**	**P_{het}**
HR (per 500 risk alles)	1.31 (1.14-1.50)	8.5×10^{-5}	1.57 (1.47-1.67)	3.1×10^{-42}	1.51 (1.43-1.61)	2.2×10^{-44}	82	0.018
ΔC	0.004 (-0.001-0.008)	0.116	0.006 (0.004-0.008)	3.7×10^{-7}	0.006 (0.003-0.008)	1.6×10^{-7}	0	0.353
NRI	0.246 (0.131-0.360)	2.8×10^{-5}	0.216 (0.136-0.296)	1.2×10^{-7}	0.226 (0.160-0.291)	1.6×10^{-11}	0	0.681
ΔAUDC (%)	1.9 (-0.5-4.4)	0.123	4.8 (1.8-7.9)	1.4×10^{-3}	3.8 (1.7-6.1)	3.3×10^{-4}	54	0.140

Est. is estimate; CI are confidence intervals; P-values are for the null hypothesis of no association with the polygenic risk score (HR=1, ΔC=0, NRI=0, ΔAUDC=0); I^2 is the proportion of variance in the parameter explained by ancestry group (an estimate of heterogeneity); P_{het} is for the null hypothesis that estimates in AfrAm and EurAm are equal. Alles stands for alleles. Models involve adjustment for age, sex, parental history of diabetes, body mass index and fasting glucose.

Table S10: Hazard Ratio (HR), Improvement in C-statistic (ΔC), Net Reclassification Information (NRI) and Improvement in Area Under Decision Curve(ΔAUDC) for PGS002771 [12]

	AfrAm		EurAm		Combined AfrAm+EurAm			
**Parameter**	**Est. (95% CI)**	**P-value**	**Est. (95% CI)**	**P-value**	**Est. (95% CI)**	**P-value**	**I^2 (%)**	**P_{het}**
HR (per 5000 risk alles)	1.17 (1.06-1.29)	1.3×10^{-3}	1.81 (1.72-1.91)	8.0×10^{-107}	1.63 (1.56-1.71)	6.6×10^{-96}	98	7.4×10^{-15}
ΔC	0.001 (-0.002-0.004)	0.693	0.011 (0.008-0.014)	1.6×10^{-11}	0.006 (0.003-0.008)	4.5×10^{-7}	95	4.5×10^{-7}
NRI	0.098 (-0.028-0.224)	0.127	0.345 (0.270-0.421)	3.2×10^{-19}	0.280 (0.215-0.345)	2.4×10^{-17}	91	9.8×10^{-4}
ΔAUDC (%)	0.8 (-1.1-2.8)	0.432	6.5 (3.0-10.1)	2.5×10^{-4}	4.6 (2.2-7.1)	1.7×10^{-4}	87	5.8×10^{-3}

Est. is estimate; CI are confidence intervals; P-values are for the null hypothesis of no association with the polygenic risk score (HR=1, ΔC=0, NRI=0, ΔAUDC=0); I^2 is the proportion of variance in the parameter explained by ancestry group (an estimate of heterogeneity); P_{het} is for the null hypothesis that estimates in AfrAm and EurAm are equal. Alles stands for alleles. Models involve adjustment for age, sex, parental history of diabetes, body mass index and fasting glucose.

Table S11. Hazard Ratios, Improvement in C-statistic (ΔC), Net Reclassification Information (NRI) and Improvement in Area Under Decision Curve(ΔAUDC) for PGS002308 (PRS) and BMI- Models without Adjustment for Fasting Glucose

	AfrAm		EurAm		Combined AfrAm+EurAm			
**Hazard Ratios**	**Est. (95% CI)**	**P-value**	**Est. (95% CI)**	**P-value**	**Est. (95% CI)**	**P-value**	**I^2 (%)**	**P_{het}**
PRS (per 5000 risk alles)	1.91 (1.74-2.10)	4.3×10^{-41}	1.99 (1.88-2.10)	9.3×10^{-131}	1.96 (1.87-2.06)	7.7×10^{-170}	0	0.470
BMI (per kg/m^2)	1.06 (1.05-1.07)	2.6×10^{-23}	1.13 (1.12-1.14)	1.1×10^{-222}	1.11 (1.10-1.11)	2.3×10^{-225}	99	1.3×10^{-20}
**ΔC**								
PRS	0.064 (0.050-0.078)	5.8×10^{-19}	0.034 (0.028-0.040)	8.9×10^{-28}	0.039 (0.033-0.044)	6.3×10^{-42}	93	1.5×10^{-4}
BMI	0.022 (0.012-0.032)	2.5×10^{-5}	0.063 (0.054-0.071)	1.0×10^{-47}	0.046 (0.040-0.053)	1.0×10^{-43}	97	1.9×10^{-9}
**NRI**								
PRS	0.494 (0.385-0.603)	7.1×10^{-19}	0.424 (0.358-0.490)	2.2×10^{-36}	0.442 (0.386-0.499)	2.6×10^{-53}	14	0.282
BMI	0.407 (0.295-0.520)	1.1×10^{-12}	0.614 (0.548-0.681)	2.2×10^{-73}	0.560 (0.503-0.618)	2.5×10^{-82}	90	1.9×10^{-3}
**ΔAUDC (%)**								
PRS	33.3 (23.0-44.4)	2.5×10^{-12}	18.2 (12.4-24.2)	4.9×10^{-11}	22.4 (15.3-30.0)	3.6×10^{-11}	84	0.013
BMI	10.1 (4.2-16.3)	6.5×10^{-4}	40.0 (31.7-48.8)	3.3×10^{-27}	33.5 (24.9-42.6)	1.2×10^{-17}	97	1.0×10^{-8}

Est. is estimate; CI are confidence intervals; P-values are for the null hypothesis of no association with the polygenic risk score (HR=1, ΔC=0, NRI=0, ΔAUDC=0); I^2 is the proportion of variance in the parameter explained by ancestry group (an estimate of heterogeneity); P_{het} is for the null hypothesis that estimates in AfrAm and EurAm are equal. Alles stands for alleles. Models involve adjustment for age, sex, parental history of diabetes, body mass index and fasting glucose.

Table S12: Hazard Ratio (HR), Improvement in C-statistic (ΔC), Net Reclassification Information (NRI) and Improvement in Area Under Decision Curve(ΔAUDC) for PGS002308 [13] with Adjustment for Study-Specific Principal Components

	AfrAm		EurAm		Combined AfrAm+EurAm			
**Parameter**	**Est. (95% CI)**	**P-value**	**Est. (95% CI)**	**P-value**	**Est. (95% CI)**	**P-value**	**I^2 (%)**	**P_{het}**
HR (per 5000 risk alles)	2.05 (1.83-2.29)	1.3×10^{-35}	1.77 (1.6-1.88)	110×10^{-88}	1.83 (1.74-1.92)	2.3×10^{-121}	80	0.026
ΔC	0.023 (0.015-0.032)	1.4×10^{-7}	0.010 (0.007-0.013)	3.5×10^{-11}	0.012 (0.009-0.014)	1.4×10^{-15}	87	5.1×10^{-3}
NRI	0.464 (0.346-0.582)	1.5×10^{-14}	0.322 (0.244-0.400)	6.3×10^{-16}	0.365 (0.300-0.431)	4.7×10^{-28}	74	0.050
ΔAUDC (%)	14.2 (8.4-20.3)	6.2×10^{-7}	5.7 (2.3-9.1)	7.9×10^{-3}	8.9 (5.8-12.0)	4.5×10^{-9}	84	0.013

Est. is estimate; CI are confidence intervals; P-values are for the null hypothesis of no association with the polygenic risk score (HR=1, ΔC=0, NRI=0, ΔAUDC=0); I^2 is the proportion of variance in the parameter explained by ancestry group (an estimate of heterogeneity); P_{het} is for the null hypothesis that estimates in AfrAm and EurAm are equal. Alles stands for alleles. Models involve adjustment for age, sex, parental history of diabetes, body mass index, fasting glucose, and the first 4 genetic principal components, derived in each cohort separately.

Table S13: Hazard Ratio (HR), Improvement in C-statistic (ΔC), Net Reclassification Information (NRI) and Improvement in Area Under Decision Curve(ΔAUDC) for PGS002308 [13] with Adjustment for Principal Components Projected from 1000 Genomes Data

	AfrAm		EurAm		Combined AfrAm+EurAm			
**Parameter**	**Est. (95% CI)**	**P-value**	**Est. (95% CI)**	**P-value**	**Est. (95% CI)**	**P-value**	**I^2 (%)**	**P_{het}**
HR (per 5000 risk alles)	2.02 (1.80-2.26)	5.8×10^{-34}	1.79 (1.69-1.89)	2.2×10^{-90}	1.83 (1.74-1.92)	9.7×10^{-122}	72	0.059
ΔC	0.022 (0.014-0.031)	1.8×10^{-7}	0.010 (0.007-0.013)	1.1×10^{-10}	0.012 (0.009-0.014)	3.9×10^{-15}	86	7.5×10^{-3}
NRI	0.446 (0.329-0.564)	9.1×10^{-14}	0.324 (0.247-0.402)	2.4×10^{-16}	0.361 (0.297-0.426)	6.6×10^{-28}	65	0.089
ΔAUDC (%)	10.7 (5.3-16.2)	5.7×10^{-5}	5.2 (1.9-8.9)	2.2×10^{-3}	7.4 (4.4-10.4)	9.3×10^{-7}	62	0.105

Est. is estimate; CI are confidence intervals; P-values are for the null hypothesis of no association with the polygenic risk score (HR=1, ΔC=0, NRI=0, ΔAUDC=0); I^2 is the proportion of variance in the parameter explained by ancestry group (an estimate of heterogeneity); P_{het} is for the null hypothesis that estimates in AfrAm and EurAm are equal. Alles stands for alleles. Models involve adjustment for age, sex, parental history of diabetes, body mass index, fasting glucose, and the first 4 genetic principal components, derived from 2504 participants in the 1000 Genomes Project and projected into each cohort[3].

Figure S1. Distribution of PGS002308 [13] by Ancestry Group with "ad hoc" Adjustment for Effects of Principal Components Projected from 1000 Genomes Data

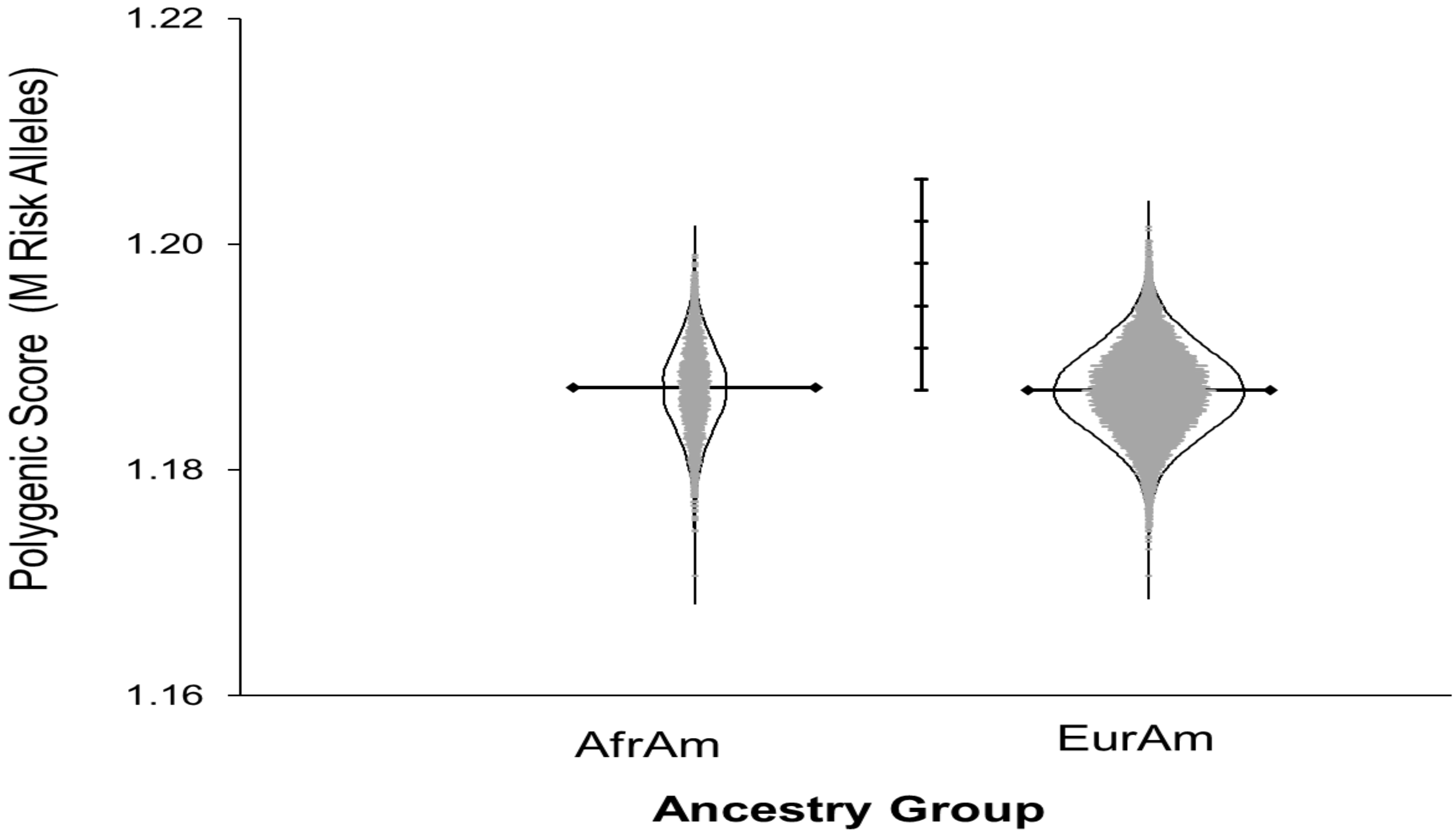

Figure S1 Legend. A. "Bean" plots of distribution of PGS002308, adjusted for principal components derived from the 1000 Genomes Project, by ancestry group. Gray horizontal lines represent the number of individuals at each level of the PRS; the thick black horizonal lines represent the mean values and the thin curved lines represent the density function. Ticks on the ruled scale represent SD units in the pooled sample.

Figure S2 Comparison of Polygenic Scores for Prediction of Incident Diabetes with Adjustment for "Overfitting"

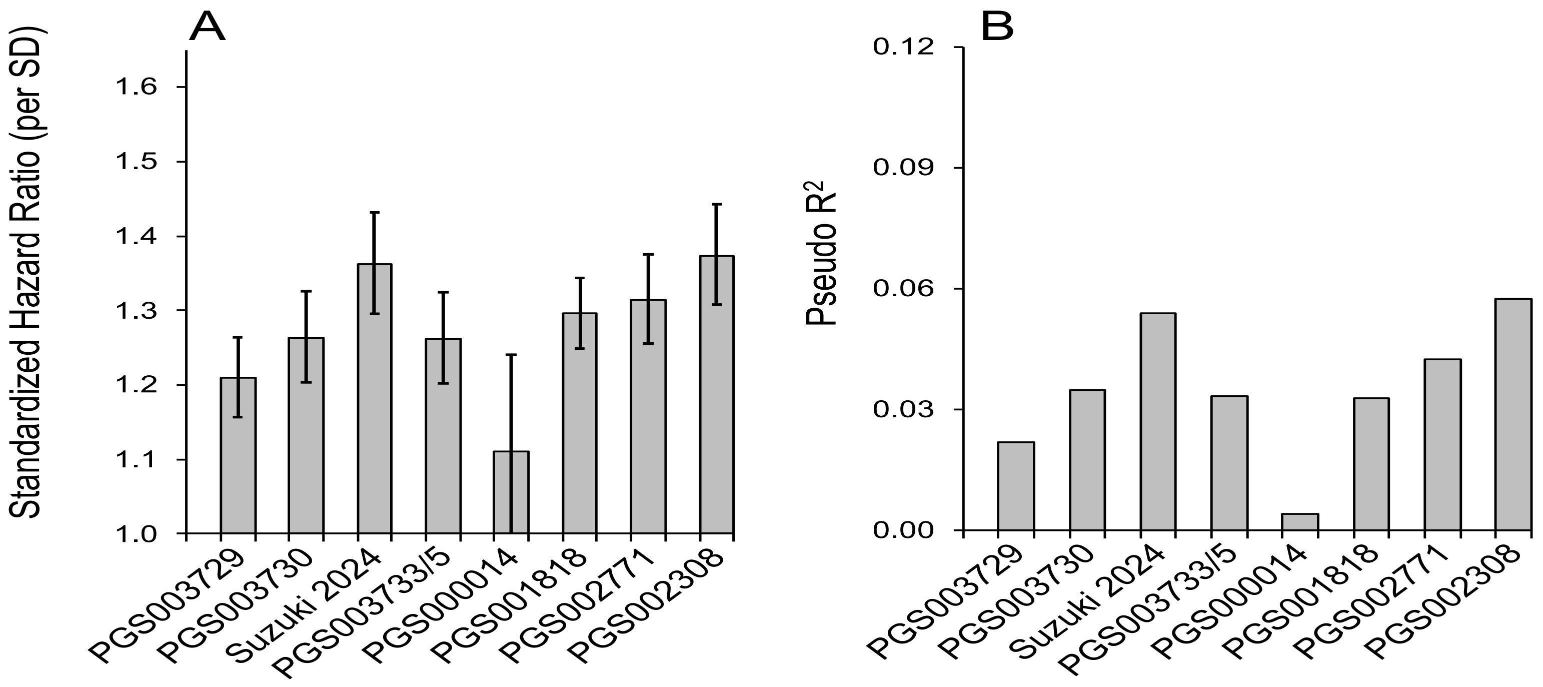

Figure S2 Legend: Strength of association, adjusted for "overfitting" by meta-regression, for each of the eight PRSs evaluated. A. Standardized hazard ratio, expressed per SD of the PRS in each cohort and ancestry group. B. Pseudo-r^2, a measure of randomness explained. See Table 1 for identity of PRSs.

Figure S3. Decision Curve Analyses forPGS002308 in AfrAm, EurAm and Combined Sample with Adjustment for "Overfitting"

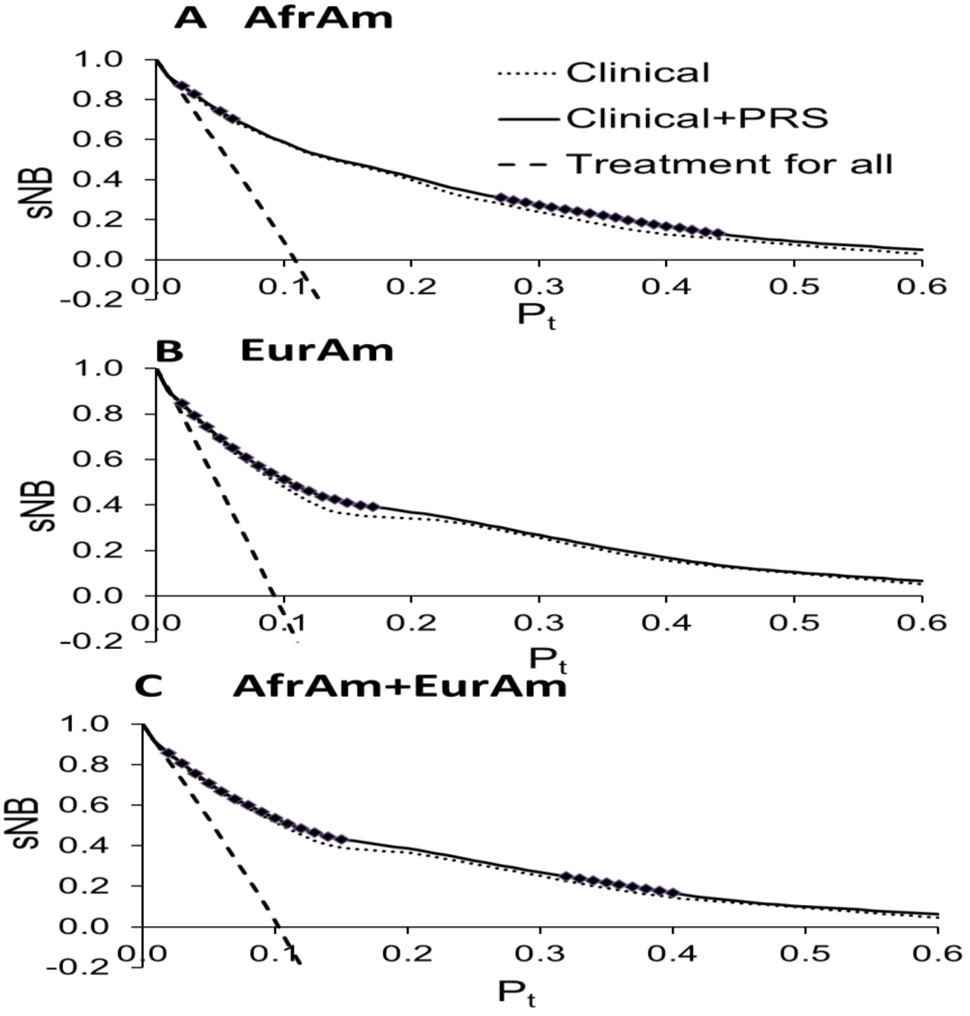

Figure S3 Legend: Decision curves, with adjustment for overfitting, for prediction of diabetes for models using clinical variables alone, clinical variables and the PRS, and for a model with treatment for all (without selection of high-risk individuals). sNB is the standardized net benefit, and p_t is the threshold probability above which individuals are selected for intervention. At p_t=0 there are no costs for false positives and sNB=1 for all curves; at higher p_t costs of false positives are higher and strategies that more accurately predict diabetes associate with higher benefit. Points where the PRS provides significantly ($p<0.05$) greater benefit than clinical variables alone are shown with diamonds. A. curves for AfrAm, B. curves for EurAm, C. curves for combined sample. Among all participants, sNB improved by 3.6% at pt=0.10, by 5.5% at pt=0.20 and by 7.0% at pt=0.30. In AfrAm corresponding values were 0.6%, 3.6% and 14.1%, while in EurAm they were 6.3%, 8.2% and 4.9%.

Figure S4.

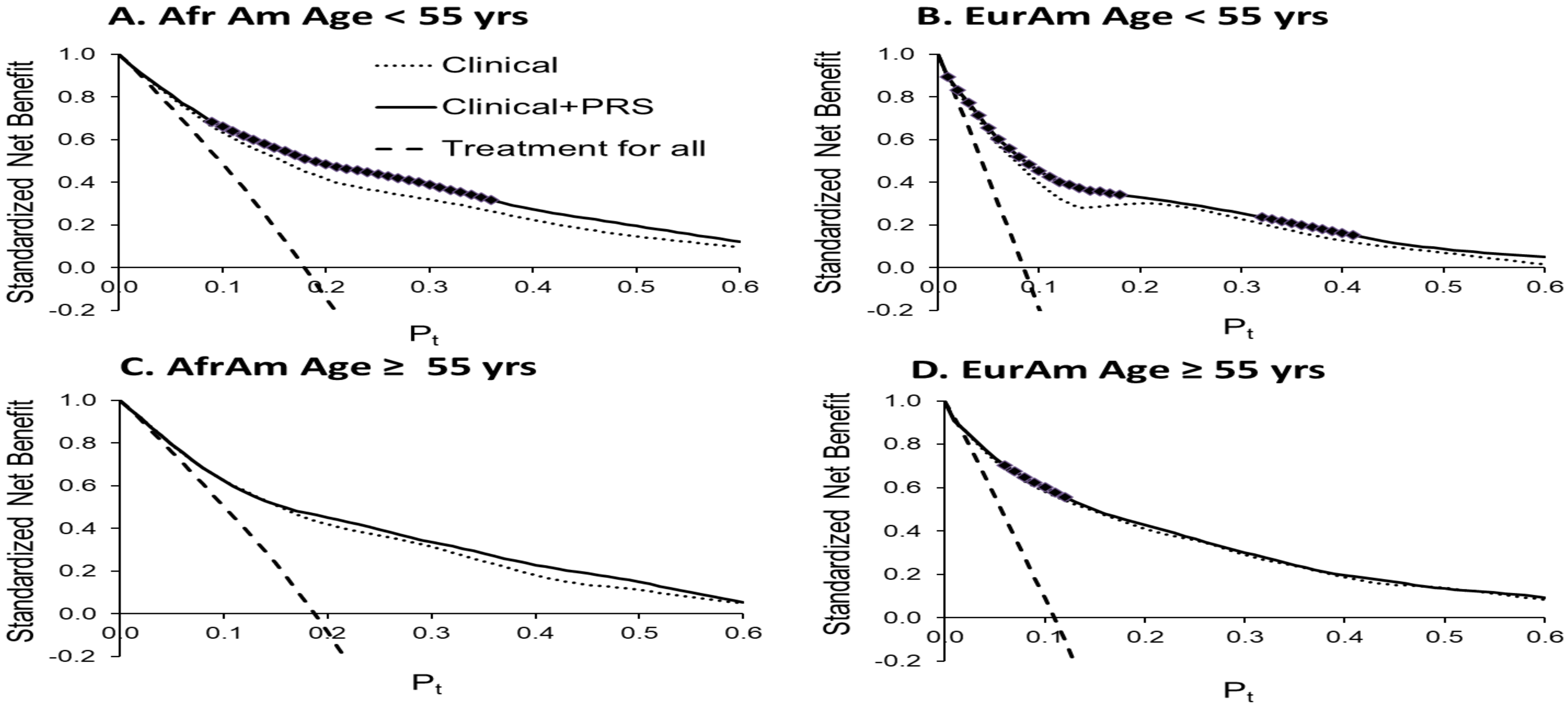

Figure S4 Legend: Decision curves for prediction of diabetes for models using clinical variables alone, clinical variables and the PRS, and for a model with treatment for all (without selection of high-risk individuals). sNB is the standardized net benefit, and p_t is the threshold probability above which individuals are selected for intervention. At $p_t=0$ there are no costs for false positives and sNB=1 for all curves; at higher p_t costs of false positives are higher and strategies that more accurately predict diabetes associate with higher benefit. Points where the PRS provides significantly ($p<0.05$) greater benefit than clinical variables alone are shown with diamonds. A. Curves for AfrAm age <55yrs, B. Curves for EurAm age <55 yrs, C. Curves for AfrAm age ≥55 yrs, D. Curves for EurAm age ≥55 yrs.

Supplement References

1. Mailman MD, Feolo M, Jin Y, Kimura M, Tryka K, Bagoutdinov R, et al. The NCBI dbGaP database of genotypes and phenotypes. Nature genetics. 2007;39(10):1181-6.
2. American Diabetes Association Expert Committee on the Diagnosis and Classification of Diabetes Mellitus. Report of the Expert Committee on the Diagnosis and Classification of Diabetes Mellitus. Diabetes Care. 1997;20(7):1183-97.
3. The 1000 Genomes Project Consortium. A global reference for human genetic variation. Nature. 2015;526(7571):68-74.
4. Browning SR, Browning BL. Rapid and accurate haplotype phasing and missing-data inference for whole-genome association studies by use of localized haplotype clustering. American journal of human genetics. 2007;81(5):1084-97.
5. Browning BL, Zhou Y, Browning SR. A One-Penny Imputed Genome from Next-Generation Reference Panels. American journal of human genetics. 2018;103(3):338-48.
6. Frazer KA, Ballinger DG, Cox DR, Hinds DA, Stuve LL, Gibbs RA, et al. A second generation human haplotype map of over 3.1 million SNPs. Nature. 2007;449(7164):851-61.
7. Mahajan A, Taliun D, Thurner M, Robertson NR, Torres JM, Rayner NW, et al. Fine-mapping type 2 diabetes loci to single-variant resolution using high-density imputation and islet-specific epigenome maps. Nature genetics. 2018;50(11):1505-13.
8. Mahajan A, Spracklen CN, Zhang W, Ng MCY, Petty LE, Kitajima H, et al. Multi-ancestry genetic study of type 2 diabetes highlights the power of diverse populations for discovery and translation. Nature genetics. 2022;54(5):560-72.
9. Suzuki K, Hatzikotoulas K, Southam L, Taylor HJ, Yin X, Lorenz KM, et al. Genetic drivers of heterogeneity in type 2 diabetes pathophysiology. Nature. 2024;627(8003):347-57.
10. Khera AV, Chaffin M, Aragam KG, Haas ME, Roselli C, Choi SH, et al. Genome-wide polygenic scores for common diseases identify individuals with risk equivalent to monogenic mutations. Nature genetics. 2018;50(9):1219-24.
11. Privé F, Aschard H, Carmi S, Folkersen L, Hoggart C, O'Reilly PF, et al. Portability of 245 polygenic scores when derived from the UK Biobank and applied to 9 ancestry groups from the same cohort. American journal of human genetics. 2022;109(1):12-23.
12. Mars N, Lindbohm JV, Della Briotta Parolo P, Widén E, Kaprio J, Palotie A, et al. Systematic comparison of family history and polygenic risk across 24 common diseases. American journal of human genetics. 2022;109(12):2152-62.
13. Ge T, Irvin MR, Patki A, Srinivasasainagendra V, Lin YF, Tiwari HK, et al. Development and validation of a trans-ancestry polygenic risk score for type 2 diabetes in diverse populations. Genome Med. 2022;14(1):70.
